# Tumour cells are sensitised to ferroptosis via RB1CC1‐mediated transcriptional reprogramming

**DOI:** 10.1002/ctm2.747

**Published:** 2022-02-27

**Authors:** Xiangfei Xue, Lifang Ma, Xiao Zhang, Xin Xu, Susu Guo, Yikun Wang, Shiyu Qiu, Jiangtao Cui, Wanxin Guo, Yongchun Yu, Fenyong Sun, Yi Shi, Jiayi Wang

**Affiliations:** ^1^ Department of Clinical Laboratory Shanghai Tenth People's Hospital of Tongji University Shanghai China; ^2^ Department of Clinical Laboratory Shanghai Chest Hospital, Shanghai Jiao Tong University School of Medicine Shanghai China; ^3^ Shanghai Institute of Thoracic Oncology Shanghai Chest Hospital, Shanghai Jiao Tong University School of Medicine Shanghai China; ^4^ Bio‐X Institutes, Key Laboratory for the Genetics of Developmental and Neuropsychiatric Disorders, Ministry of Education Shanghai Jiao Tong University Shanghai China

**Keywords:** drug screening, ELP3‐mediated histone modification, enhancer, nuclear translocation, Rb1cc1 knockout mice, ROS

## Abstract

**Background:**

Ferroptosis, a form of regulated cell death, is an important topic in the field of cancer research. However, the signalling pathways and factors that sensitise tumour cells to ferroptosis remain elusive.

**Methods:**

We determined the level of ferroptosis in cells by measuring cell death and lipid reactive oxygen species (ROS) production. The expression of RB1‐inducible coiled‐coil 1 (RB1CC1) and related proteins was analyzed by immunoblotting and immunohistochemistry. Immunofluorescence was used to determine the subcellular localization of RB1CC1. We investigated the mechanism of RB1CC1 nuclear translocation by constructing a series of RB1CC1 variants. To examine the ferroptosis‐ and RB1CC1‐dependent transcriptional program in tumour cells, chromatin immunoprecipitation sequencing was performed. To assess the effect of c‐Jun N‐terminal kinase (JNK) agonists on strenthening imidazole ketone erastin (IKE) therapy, we constructed cell‐derived xenograft mouse models. Mouse models of hepatocellular carcinoma to elucidate the importance of Rb1cc1 in IKE‐based therapy of liver tumourigenesis.

**Results:**

RB1CC1 is upregulated by lipid ROS and that nuclear translocation of phosphorylation of RB1CC1 at Ser537 was essential for sensitising ferroptosis in tumour cells. Upon ferroptosis induction, nuclear RB1CC1 sharing forkhead box (FOX)‐binding motifs recruits elongator acetyltransferase complex subunit 3 (ELP3) to strengthen H4K12Ac histone modifications within enhancers linked to ferroptosis. This also stimulated transcription of ferroptosis‐associated genes, such as coiled‐coil–helix–coiled‐coil–helix domain containing 3 (CHCHD3), which enhanced mitochondrial function to elevate mitochondrial ROS early following induction of ferroptosis. FDA‐approved JNK activators reinforced RB1CC1 nuclear translocation and sensitised cells to ferroptosis, which strongly suggested that JNK is upstream of RB1CC1. Nuclear localisation of RB1CC1 correlated with lipid peroxidation in clinical lung cancer specimens. Rb1cc1 was essential for ferroptosis agonists to suppress liver tumourigenesis in mice.

**Conclusions:**

Our findings indicate that RB1CC1‐associated signalling sensitises tumour cells to ferroptosis and that targeting RB1CC1 may be beneficial for tumour treatment.

## Graphical abstract

- Nuclear translocation of RB1CC1 sensitises tumour cells to ferroptosis.
- Phosphorylation at S537 by JNK is prerequisite for RB1CC1 to translocate into nucleus.
- RB1CC1 recruits ELP3 to reinforce H4K12Ac within ferroptosis‐associated enhancers, and RB1CC1 activates transcriptional reprogramming to enhance mitochondrial function.
- RB1CC1 is an indispensible target for ferroptosis‐based anti‐tumour therapy

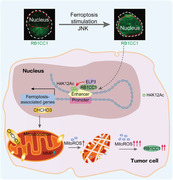

## 1 BACKGROUND

Ferroptosis, a form of regulated cell death that was identified in 2012,^1^ is characterised by mitochondrial changes resulting from excessive iron‐dependent accumulation of intracellular lipid reactive oxygen species (ROS).^2^, ^3^ Because of an increased cell metabolism and accompanied ROS generation, induction of ferroptosis is highly sensitive in a variety of tumour cells.^4^, ^5^ Triggering ferroptosis can be effective in tumour cells resistant to pro‐apoptotic and other conventional anti‐tumour treatments.^5^, ^6^ Studying ferroptosis has therefore becoming a new hot spot in the field of tumour research.

Although inhibiting tumour growth by the induction of ferroptosis is a promising strategy for tumour treatment, tumour cells may alter their sensitivities to ferroptosis via various gene expression and regulation mechanisms.^7^, ^8^ For example, upregulation of solute carrier family 7 member 11 (SLC7A11), the functional subunit of system X_C_^–^, and an antioxidant transcription factor nuclear factor, erythroid 2‐like 2 (NFE2L2 or known as NRF2), results in a ferroptosis‐resistant state in cells.^9^, ^10^ Emerging evidence has also demonstrated that changes in signal transduction pathways may play a key role in ferroptosis resistance.^4^, ^6^, ^10^ Comprehensively studying the signalling networks and involved factors underlying ferroptosis is critical to develop an effective strategy to sensitise tumour cells to ferroptosis and to develop effective ferroptosis‐based treatments.

Ferroptosis is regulated and coordinated by various factors involving changes in iron metabolism, redox system and lipid peroxidation.^2^, ^11^ Notably, any of these factors is indispensable for occurrence of ferroptosis.^3^ Iron is an essential factor for ferroptosis, as it participates in the formation of free radicals and lipid ROS production.^12^ Abnormal regulation of iron‐related proteins, such as transferrin (TRF), transferrin receptor 1 (TfR1) and ferritin, can cause iron overload and sensitise tumour cells to ferroptosis.^13^ By contrast, heat shock protein family B member 1 (HSPB1)‐, protein kinase C (PKC)‐ and Kelch‐like epichlorohydrin (ECH)‐associated protein1 (KEAP1)/NRF2‐linked signalling can block iron absorption, thus preventing ferroptosis.^10^, ^14^, ^15^ The redox system also plays a critical role in ferroptosis. Glutathione (GSH) is the most abundant intracellular antioxidant, and glutathione peroxidases (GPXs) use GSH as a co‐factor to reduce lipid peroxide. Suppressing GSH synthesis and inactivating GPX4 can both induce ferroptosis.^4^, ^16^ The core process in ferroptosis is an excessive accumulation of lipid ROS.^2^, ^17^ Compared with the effects of iron and redox system on ferroptosis, the effects of lipid metabolism on ferroptosis are more complicated. Although the enzymes responsible for lipid remodelling have been identified,^6^, ^18^ the factors regulated by lipid ROS have not been thoroughly elucidated, and whether targeting such factors increases the efficacy of pro‐ferroptotic treatment also remains unclear.

In this work, we aimed to investigate lipid ROS‐regulated signalling and identify targets to sensitise tumour cells to ferroptosis. RB1‐inducible coiled‐coil 1 (RB1CC1, also known as FIP200) was identified to be positively regulated by lipid ROS. RB1CC1 is the basic autophagy factor for the formation of autophagosomes.^19^ RB1CC1 is also linked to cell proliferation and the cell cycle.^20^, ^21^ RB1CC1 has distinct functions depends on its subcellular localisation.^22^, ^23^ We discovered that nuclear translocation of RB1CC1 upon induction of ferroptosis triggers transcriptional reprogramming, which is essential for increasing the sensitivity of tumour cells to ferroptosis. Our study reveals a novel signalling pathway that can be targeted to reinforce the efficacy of pro‐ferroptotic treatment against tumours.

## 2 METHODS

### 2.1 Cell culture

HepG2, MKN45, H1299, PC9, A549, H226, SW1990, Bel‐7402, HCT29, H460, Calu‐1 and HEK293T cell lines were purchased from Fuheng Biotechnology (Shanghai, China). The cell lines were validated by short tandem repeat analysis and verified to be mycoplasma‐free lines. The 143B RHO° cells and parental 143B cells were kindly provided by Prof. Hezhi Fang (Wenzhou Medical University, Wenzhou, China). Mouse embryonic fibroblasts (MEFs) were established from mouse embryos at E13.5. Patient‐derived primary lung squamous cell carcinoma (LUSC) cells were generated from LUSC tissues as described in a prior study.^24^ All cells except 143B RHO° cells were maintained in Dulbecco's modified eagle's medium (DMEM, SH30243.1, HyClone, Logan, UT, USA) supplemented with 10% fetal bovine serum (FBS), 100 U/ml penicillin and 100 mg/ml streptomycin. The 143B RHO° cells were maintained in DMEM containing 50 μg/ml uridine (U3003, Sigma, St. Louis, MO, USA).

### 2.2 Reagents and plasmids

For reagents, dimethylsulfoxide (DMSO, ST038, Beyotime, Shanghai, China), erastin (E7781, Sigma), ferrostatin‐1 (Fer‐1, SML0583, Sigma), RSL3 (S8155, Selleckchem, Shanghai, China), deferoxamine (DFO, Y0001937, Sigma), imidazole ketone erastin (IKE, HY‐114481, MCE, Shanghai, China), liproxstatin‐1 (Lipro‐1, HY‐12726, MCE), Earle's Balanced Salt Solution (EBSS, C0213, Beyotime), inhibitor‐1 of trichostatin A (ITSA, S8323, Selleckchem), carbonyl cyanide 3‐chlorophenylhydrazone (CCCP, C2006, Beyotime), wortmannin (Wort, HY‐10197, MCE), paclitaxel (PTX, S1150, Selleckchem), clofarabine (Clolar, S1218, Selleckchem), oxaliplatin (OXA, S1224, Selleckchem), temozolomide (TMZ, S1237, Selleckchem), JNK‐IN‐8 (HY‐13319, MCE), SP600125 (HY‐12041, MCE), cisplatin (HY‐17394, MCE), diethylnitrosamine (DEN, N0756, Sigma) and carbon tetrachloride (CCl_4_, 1601168, Sigma) were used for cell culture and animal experiments. The Food and Drug Administration (FDA)‐approved drug library was purchased from Selleckchem (L1300) and used for drug screening. Cell fractionation experiments were performed using a nuclear and cytoplasmic protein extraction kit (P0027, Beyotime). LentiCRISPR v2‐based plasmid constructs were used to knockout (KO) RB1CC1, coiled‐coil–helix–coiled‐coil–helix domain containing 3 (CHCHD3) and elongator acetyltransferase complex subunit 3 (ELP3). RB1CC1‐ and CHCHD3‐expressing plasmids were constructed using the pCMV plasmid. siRNAs targeting TPBG, EPRS1, NAMPT, FOSL1, LZTFL1 and RB1CC1 were purchased from GenePharma Co., Ltd. (Shanghai, China). The green fluorescent protein (GFP)‐LC3 plasmid was purchased from Shengyang Biotech (Shanghai, China). The pcDNA3.1(+)‐based plasmids expressing RWT‐, RΔ1‐, RΔ2‐, RΔ3‐, RΔ4‐, RΔ5‐, RΔ6‐RB1CC1‐FLAG, RB1CC1^ΔC^‐FLAG, ELP3^WT^‐MYC and ELP3^ΔC^‐MYC were constructed using routine polymerase chain reaction (PCR). The pcDNA3.1(+)‐based plasmids expressing Y513A‐, Y528A‐, S533A‐, S537A‐, S545A‐ and S537E‐RB1CC1‐FLAG were constructed using overlap PCR. Detailed primers and siRNA sequences are listed in Table S1.

### 2.3 Animal experiments

To evaluate the role of Rb1cc1 in lipid peroxidation and regulation of Chchd3 expression, *Rb1cc1^+/–^* mice were interbred with each other, and the foetuses were obtained from pregnant female mice at E19 by cesarean section following pre‐administration with IKE (50 mg/kg), with or without Lipro‐1 (10 mg/kg) for 2 days.

To evaluate the effects of drugs to strengthen the effects of IKE, cell‐derived xenograft (CDX) mouse models were generated by subcutaneously injecting 2 × 10^6^ cells per athymic nude mouse (BALB/c‐nu, Spaefer, Beijing, China). When tumours were 220–250 mm^3^, mice were administered with IKE (50 mg/kg) daily with or without PTX (20 mg/kg), Clolar (10 mg/kg), OXA (10 mg/kg) or TMZ (40 mg/kg). Tumour growth was monitored, and sizes were calculated by 0.5 × *L* × *W* ^2^ (*L* indicating length and *W* indicating width).

To clarify the importance of Rb1cc1 for the IKE‐based therapy in liver tumourigenesis, wild‐type (*WT)* and *Rb1cc1^+/–^* mice were intraperitoneally injected with DEN (25 mg/kg) at 2 weeks post‐birth followed by injection with CCl_4_ (0.5 mg/kg) from the 6th to 21st week post‐birth for a total of 16 times. IKE (50 mg/kg) was regularly administered every 2 days from the 9th to the 21st week post‐birth. All mice were sacrificed at the 30th week post‐birth. All mouse experiments were approved by the institutional ethics committee of Shanghai Chest Hospital.

### 2.4 Quantitative reverse transcription PCR (qRT‐PCR), immunoblotting (IB) and immunohistochemistry (IHC)

For qRT‐PCR, total RNA was extracted using an RNA‐easy isolation reagent (R701‐02, Vazyme, Nanjing, China) and then reverse transcribed into cDNA using the PrimeScript™ RT‐PCR kit (RR014A, Takara, Dalian, China). qRT‐PCR was performed using Premix Ex Taq™ (RR390A, Takara) to evaluate the mRNA expressions of TPBG, EPRS1, NAMPT, FOSL1, LZTFL1, RB1CC1, RB1, CHCHD3, VRK3, TPD52, SLC25A32, MYC, CYTH3, MEPCE, CYREN and SEC3; GAPDH mRNA was used as an internal control. The primers are listed in Table S1.

IB was performed following standard protocols. The samples were resolved by electrophoresis on polyacrylamide gel electrophoresis (PAGE) gels with or without Phostag™ reagent (AAL‐107, Wako, Osaka, Japan). The primary antibodies were anti‐RB1CC1 (#12436, Cell Signaling Technology (CST), Boston, MA, USA), anti‐β‐tubulin (GB122667, Servicebio, Wuhan, China), anti‐histone H3 (#14269, CST), anti‐GAPDH (#5174, CST), anti‐FLAG (#14793, CST), anti‐GFP (#2956, CST), anti‐CHCHD3 (ab224565, Abcam, Cambridge, UK), anti‐LC3 (GB11124, Servicebio), anti‐c‐Jun N‐terminal kinase (JNK, ab179461, Abcam) and anti‐p‐JNK (#4668, CST). The membranes were incubated with secondary antibodies (#7074 or #7076, CST) and the bands were visualised with enhanced chemiluminescence (ECL) substrate (AP34L025, Life‐iLab, Shanghai, China).

For IHC, slides were stained with anti‐RB1CC1 (#12436, CST), anti‐4 hydroxynonenal (4‐HNE, ab46545, Abcam) and anti‐CHCHD3 (ab224565, Abcam) primary antibodies. Signals were visualised using diaminobenzidine (DAB) reagent (P0203, Beyotime). The lung cancer tissues included in the tissue microarray were obtained from Shanghai Chest Hospital; patient information is listed in Table S2.

### 2.5 In situ proximity ligation assay (PLA)

PLA was performed using the Duolink^®^ In Situ Red Starter Kit (mouse/rabbit, Sigma) strictly in accordance with the manufacturer's instructions. Cells seeded on glass coverslips were incubated overnight at 4°C with primary antibodies. The primary antibodies used were as follows: anti‐RB1CC1 (MABC128, Merck, Darmstadt, Germany), anti‐CHCHD3 (ab224565, Abcam), anti‐FLAG (#8146, CST) and anti‐MYC (#2272, CST). The samples were visualised using a laser scanning confocal microscope (LSM 800, CarlZeiss, Jena, Germany).

### 2.6 Luciferase reporter assay

RB1CC1 and H4K12Ac co‐peaks were PCR‐amplified and cloned into the pGL3 promoter plasmids. Reporter plasmids were co‐transfected with the Renilla luciferase plasmid into cells. The luciferase activities in cells with different treatments were measured using a Dual‐Luciferase^®^ reporter assay system (Promega, Madison, WI, USA). The mutant enhancers were synthesised by GenePharma Co., Ltd. and cloned into the pGL3 promoter plasmids.

### 2.7 Chromatin immunoprecipitation (ChIP) and Re‐ChIP

ChIP and Re‐ChIP experiments were performed using kits from Active Motif (Carlsbad, CA, USA). For ChIP, approximately 2 × 10^7^ cells were fixed in 1% formaldehyde and then sonicated. Coupled DNA was eluted in 1% sodium dodecyl sulfate (SDS)/0.1 M NaHCO_3_ and then reverse cross‐linked at 65°C. Samples were purified via phenol/chloroform extraction and ethanol precipitation and then subjected to semi‐ or real‐time‐qRT‐PCR.

For Re‐ChIP experiments, the complexes immunoprecipitated after ChIP using the first primary antibodies were eluted by incubation at 37°C in 10 mM dithiothreitol (DTT) for 30 min. After centrifugation, the supernatant was diluted 20 times with Re‐ChIP buffer (1% Triton X‐100, 2 mM ethylenediaminetetraacetic acid (EDTA), 150 mM NaCl, 20 mM Tris–HCl, pH 8.1). The sample was then examined again by ChIP with antibodies targeting a second protein. The antibodies used in ChIP and Re‐ChIP experiments were anti‐RB1CC1 (#12436, CST), anti‐H4K12Ac (ab177793, Abcam), anti‐H4K16Ac (ab109463, Abcam), anti‐FOXI1 (ab20454, Abcam), anti‐ELP3 (ab190907, Abcam) and anti‐immunoglobulin G (IgG, #3900, CST). Primers are listed in Table S1.

### 2.8 Chromosome conformation capture (3C) assay

Cross‐linking of cells was performed by incubating 1 × 10^7^ cells in phosphate buffer solution (PBS) containing 1% formaldehyde; glycine (0.125 M) was added to stop the reaction. Complexes were precipitated by ethanol at −20°C. Precipitates were dissolved in Tris–EDTA buffer to a total volume of 80 μl and digested by Alui (FD0014, Thermo Fisher, MA, USA) at 37°C for 1 h. Alui was inactivated at 65°C for 20 min and then samples were incubated with T4 DNA ligase (Promega) at 16°C for 5 h. Protein–DNA complexes were de‐cross‐linked by adding SDS and NaCl up to 1% and 0.3 M, respectively, at 55°C overnight. Samples were then treated with proteinase K at 65°C for 1 h. DNA was purified by a kit from TIANGEN Biotech (Beijing, China). To visualise the enhancer–promoter interaction, the purified DNA was PCR‐amplified. Each of the purified PCR products was sequenced to confirm the identity. Primers are listed in Table S1.

### 2.9 Measurements of cell death, lipid ROS generation, mitochondrial ROS (mitoROS) production and mitochondrial membrane potential (MMP)

To measure cell death, cells were stained by SYTOX^®^ Green (S11368, Invitrogen, Carlsbad, CA, USA) to a final concentration of 1 μM for 20 min and then analysed by flow cytometry. Lipid ROS generation was measured by adding C11‐BODIPY 581/591 (D3861, Invitrogen) to a final concentration of 1.5 μM for 30 min before flow cytometry. MitoROS production was evaluated by incubating cells with MitoSOX^®^ red mitochondrial superoxide indicator (M36008, Thermo Fisher) to a final concentration of 5 μM for 10 min before flow cytometry. JC‐1 (C2006, Beyotime) was used to indicate changes of MMP, as measured by flow cytometry.

### 2.10 Multi‐omics and bioinformatics

The proteomics experiments were performed by Oebiotech (Shanghai, China). The immunoprecipitates using anti‐RB1CC1 (#12436, CST) or anti‐Histone H4 (ab177840, Abcam) antibodies were subjected to electrophoresis; the protein bands in the Coomassie Brilliant Blue‐stained gel were excised and in‐gel digested with trypsin. The tryptic peptide digests were analysed using capillary electrophoresis/nano‐liquid chromatography systems coupled with an electrospray ionisation and quadrupole‐time‐of‐flight mass spectrometer (ESI‐QTOF‐MS, Bruker Daltonics, Leipzig, Germany). An internal MASCOT 2.4.1 server (http://www.matrixscience.com/) using the Swiss‐Prot database was employed to identify peptides. The mass spectrometry (MS) for RB1CC1 phosphorylation was performed by Shanghai Applied Protein Technology Co. Ltd (Shanghai, China). The RNA‐seq experiments were performed by Biozeron (Shanghai, China) and Oebiotech, respectively. The ChIP‐Seq experiments were also performed by Oebiotech. For a given sample, the pair‐end fastq sequencing files were first mapped to the reference genome hg38 using bowtie2, and macs3 was used to call both the broad peaks and narrow peaks according to the previous studies.^25^

**FIGURE 1:**
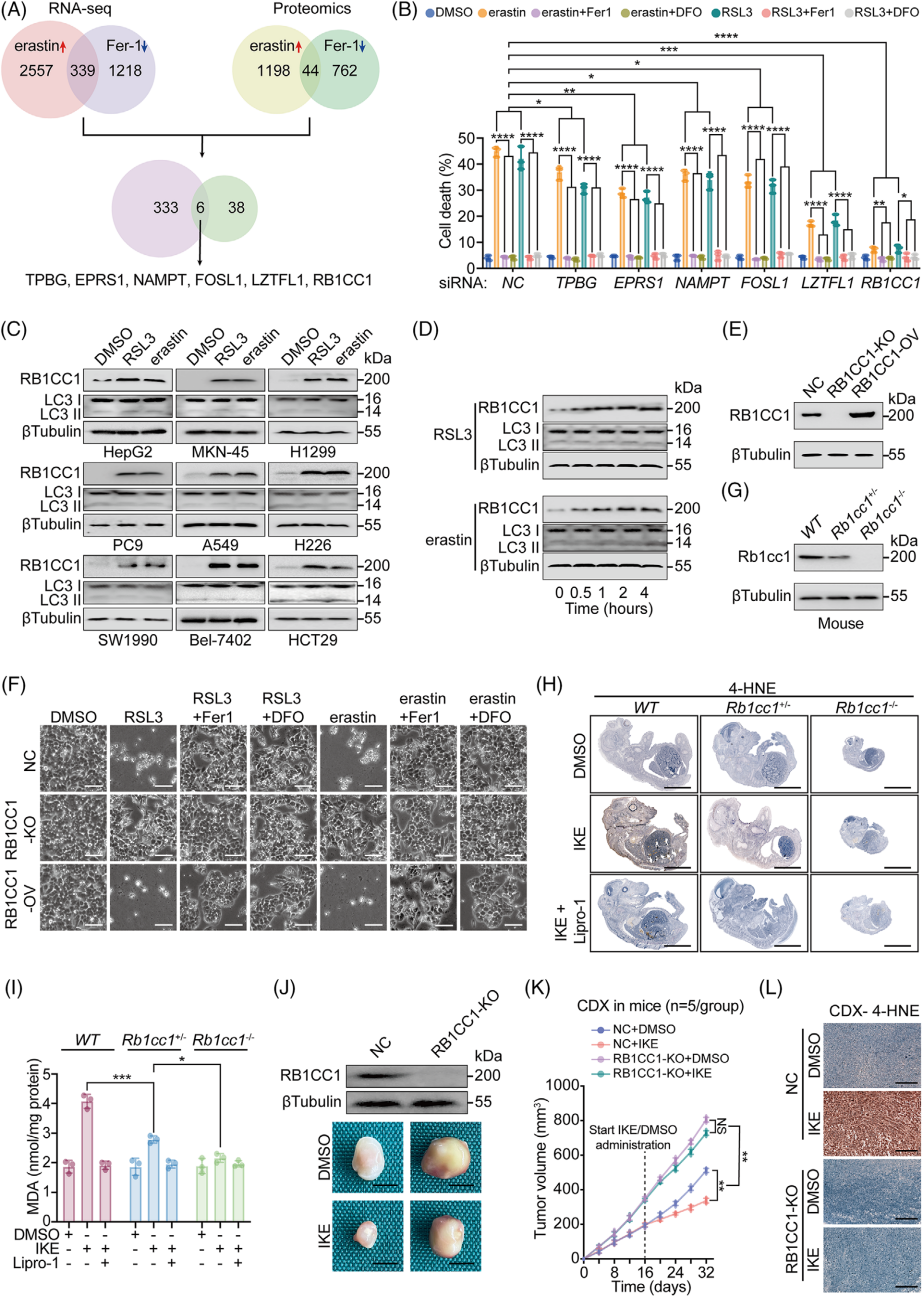
RB1CC1 sensitises tumour cells to ferroptosis. (A) Venn diagram showing RNA‐seq and proteomics results in HepG2 cells treated with erastin (10 μM) or Fer‐1 (2 μM) for 4 h. (B) Cell death, as measured by SYTOX green staining followed by cytometry in HepG2 cells transfected with siRNA, as indicated. HepG2 cells were also treated with DMSO, erastin (10 μM) or RSL3 (1 μM), with or without of Fer‐1 (2 μM) or DFO (100 μM) for 12 h. (C) IB of RB1CC1 and LC3 expression in a serial of tumour cells, as indicated, following treating with DMSO, RSL3 (1 μM) or erastin (10 μM) for 4 h. (D) IB of RB1CC1 and LC3 in HepG2 cells following treating with RSL3 (1 μM) or erastin (10 μM) for the indicated hours. (E) RB1CC1 expression efficiency for KO or overexpression (OV) of RB1CC1 in HepG2 cells. (F) Morphological changes of HepG2 cells following treating with DMSO, RSL3 (1 μM) or erastin (10 μM), with or without Fer‐1 (2 μM) or DFO (100 μM) for 12 h. Scale bar, 75 μm. (G) Rb1cc1 expression in *WT*, *Rb1cc1^+/–^* and *Rb1cc1^–/‐^* mouse foetuses. (H and I) 4‐HNE (H) and malondialdehyde (MDA) (I) in *WT*, *Rb1cc1^+/–^* and *Rb1cc1^–/‐^* mouse foetuses obtained from cesarean section at E19 following pre‐administration with IKE (50 mg/kg), with or without liproxstatin‐1 (Lipro‐1) (10 mg/kg) in pregnant female mice for 2 days. Scale bar, 3 mm. (J–L) Representative images (J), tumour growth curve (K) and 4‐HNE concentration (L) of cell‐derived xenograft (CDX) that generated by Bel‐7402 cells with or without RB1CC1 KO, and mice were administered with DMSO or IKE (50 mg/kg). Scale bar, 2 mm (J) and 200 μm (L). Statistical analysis was performed by one‐way analysis of variance (ANOVA) (B), Student's *t*‐test (I) or two‐way ANOVA (K). Data are presented as means ± SD from indicated samples. ****p < .0001, ***p < .001, **p < .01, *p < .05, indicates statistical significance and N.S. indicates non‐significance

For gene ontology (GO), enrichment analysis was performed on biological processes and cell components by the Database for Annotation, Visualization and Integrated Discovery (https://david.ncifcrf.gov/). The expression of RB1CC1 in normal and tumour tissue was analysed using the UALCAN database.

### 2.11 Measurements of cell proliferation and caspase 3/7 activities

Cell proliferation was evaluated using a BeyoClick™ EdU Cell Proliferation Kit (C0071S, Beyotime). Briefly, the cells were plated in a 24‐well plate and cultured for the indicated times, and then the cells were incubated in labelled medium containing EdU for 2 h. After removing the medium, cells were fixed for 15 min and permeabilised for 1 h. EdU was detected and the cell proliferation rate was calculated.

Caspase 3/7 activities were measured using the caspase‐Glo^®^ 3/7 assay system (G8091, Promega) in accordance with the manufacturer's instructions.

### 2.12 Statistical analysis

Statistical analysis was performed with GraphPad Prism software. All data are presented as the mean ± SD from at least three independent experiments. Differences between groups were examined by Student's *t*‐test, one‐way, two‐way ANOVA and *χ* ^2^‐test, whichever applicable. *p* < .05 was considered statistically significant. Survival curves were compared with the log‐rank test.

## 3 RESULTS

### 3.1 RB1CC1 sensitises tumour cells to ferroptosis

To screen potential lipid ROS‐regulated and ferroptosis‐associated factors, we performed RNA‐seq and proteomics in hepatocellular carcinoma (HCC) HepG2 cells, which we used in our previous study on ferroptosis regulation (Table S3 and S4 and^26^). Analyses were performed in cells treated with or without erastin, a system X_C_^–^ inhibitor that induces lipid ROS and ferroptosis, and Fer‐1, a lipid ROS scavenger that blocks ferroptosis.^1^, ^11^ From the RNA‐seq and proteomics analysis, we obtained six candidate molecules. We found that all six candidates were induced in response to erastin and reduced in response to Fer‐1 (Figure 1A), indicating they may be associated with lipid ROS generation and ferroptosis.

HepG2 cells became less sensitive to cell death induced by erastin and RSL3, another ferroptosis inducer exerting its roles via inhibiting GPX4, following silencing the six genes separately. Notably, silencing RB1CC1 resulted in the most salient effect (Figures 1B and S1A). We confirmed that the cell death was ferroptotic because cell death was blocked by not only Fer‐1, but also DFO, an iron chelator that inhibits ferroptosis (Figure 1B and^2^).

We next evaluated the expression of RB1CC1 in a series of cancer cell lines treated with RSL3 and erastin. We found a general increase of RB1CC1 in response to RSL3 and erastin in HepG2, gastric cancer MKN‐45, lung adenocarcinoma (LUAD) H1299, PC9 and A549, LUSC H226, pancreatic cancer (PACA) SW1990, HCC Bel‐7402 and colon adenocarcinoma HCT29 cells (Figures 1C and S1B). We further found that RB1CC1 was upregulated by RSL3 and erastin in a time‐dependent manner (Figures 1D and S1C). These results strongly suggested that RB1CC1 correlates with ferroptosis in tumour cells.

Previous studies indicated that RB1CC1 is involved in autophagy.^19^, ^23^ IB revealed no significant changes of LC3 II/LC3 I ratios in tumour cells treated with RSL3 and erastin and untreated cells (Figures 1C and S1B). Moreover, the LC3 II/LC3 I ratios were unaffected up to 4 h after cells were treated with RSL3 and erastin (Figures 1D and S1C). Immunofluorescence (IF) revealed that autophagosomes were induced in HepG2 cells incubated with EBSS, which triggers autophagy,^27^ and such effects were blocked by Wort, an autophagy inhibitor (Figure S1D,E). However, no significant enhancement of autophagosome formation was observed in parallel with the increase of RB1CC1 in HepG2 cells treated with RSL3 and erastin (Figure S1D,E). These results suggested that RB1CC1 sensitises ferroptosis through an autophagy‐independent manner under our experimental condition.

To address any non‐specific effects from siRNA‐mediated gene silencing, CRISPR‐Cas9‐mediated KO of RB1CC1 was performed. RB1CC1 KO desensitised HepG2 cells to ferroptosis (Figures 1E,F and S1F). Excessive accumulation of lipid ROS is a hallmark of ferroptosis.^16^, ^17^ The lipid ROS induced by RSL3 and erastin was remarkably reduced following RB1CC1 KO (Figure S1G). By contrast, ectopic OV of RB1CC1 led to increased lipid ROS (Figures 1E,F and S1G).

To explore the role of RB1CC1 in vivo, we attempted to generate mice with homozygous deletion of *Rb1cc1*. However, *Rb1cc1* null mice (*Rb1cc1* ^–/–^) were not viable or died shortly after birth. Thereby, we evaluated foetuses at E19 obtained from mice by cesarean section following pre‐administration with IKE, a more in vivo stable erastin derivative, for 2 days. The *Rb1cc1* ^–/–^ mouse foetuses were much smaller than the *WT* and *Rb1cc1* ^+/‐^ foetuses; however, no significant differences in gross phenotype were identified between *WT* and *Rb1cc1* ^+/‐^ foetuses (Figure 1G,H). Malondialdehyde and 4‐HNE are critical end products of lipid peroxidation. The IKE‐mediated induction of malondialdehyde and 4‐HNE in *Rb1cc1* ^+/–^ foetuses was not as strong as that in *WT* foetuses. However, such induction was absent in *Rb1cc1* ^–/–^ foetuses (Figure 1H,I), further demonstrating that ferroptosis‐associated lipid peroxidation is Rb1cc1 dependent.

We next examined whether RB1CC1 is essential for IKE‐induced suppression of tumour growth. CDX mouse models were generated from control and RB1CC1‐KO Bel‐7402 cells. We found that RB1CC1 KO resulted in an accelerated CDX growth. Furthermore, IKE did not suppress tumour growth and induce lipid peroxidation in the RB1CC1 KO group as it did in the control group (Figure 1J–L).

To investigate why RB1CC1 KO tumours grew faster than control cells even without IKE treatment, we compared the cell proliferation capacity of control and RB1CC1‐KO Bel‐7402 cells and found that RB1CC1 KO cells demonstrated a more accelerated cell proliferation rate than the control cells (Figure S1H). Furthermore, deficiency of RB1CC1 causes a defect in autophagy.^28^ We thereby speculated that the faster growth of RB1CC1 KO tumours are the combined results from increased cell proliferation and decreased autophagy. The above results also suggest that RB1CC1 is essential for the restriction of tumour growth and sensitisation to ferroptosis. Together these findings support the potential involvement of RB1CC1 in the regulatory signalling of ferroptosis.

### 3.2 Nuclear translocated RB1CC1 acts as a ferroptosis regulating transcription factor

**FIGURE 2:**
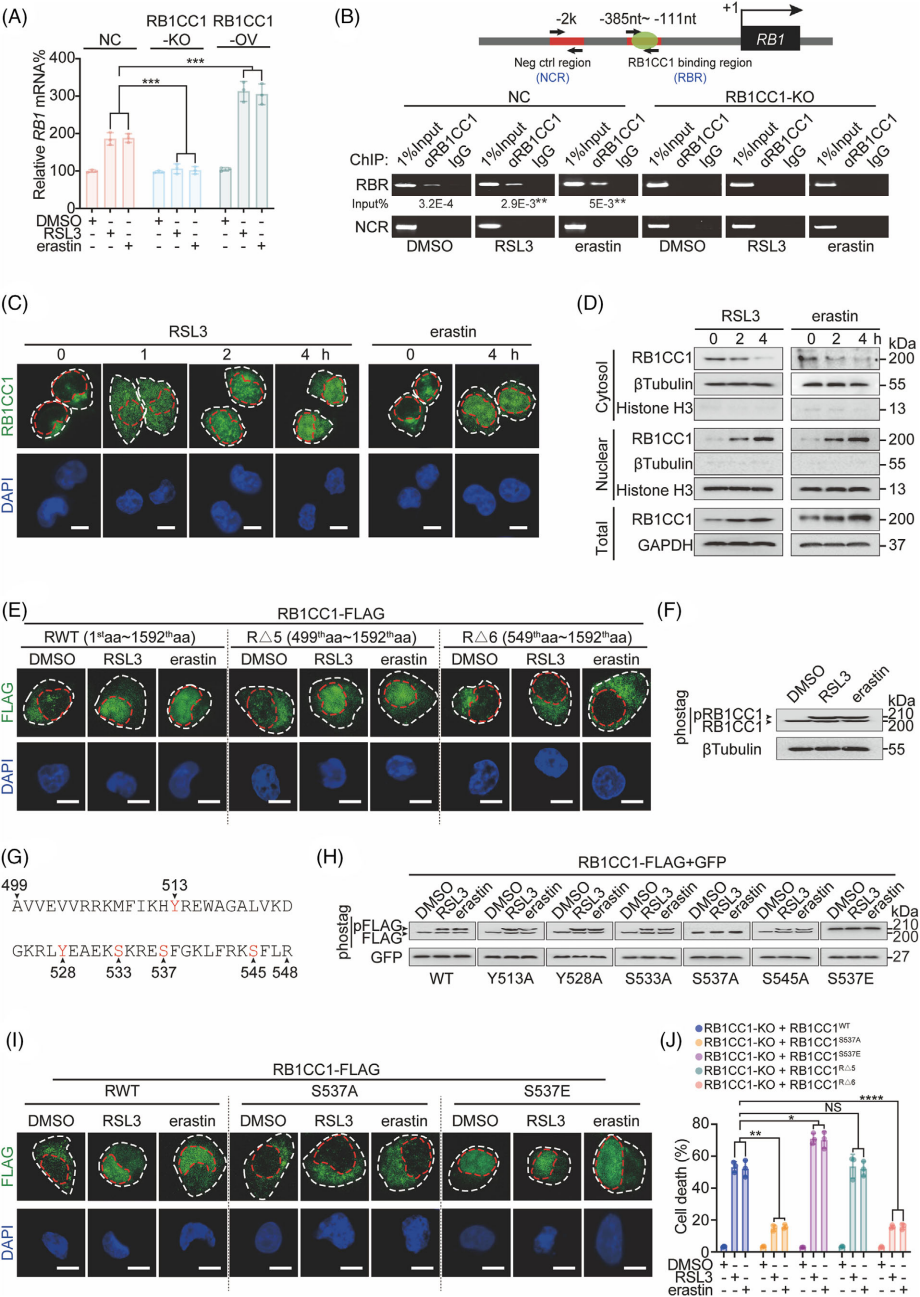
Nuclear translocation of RB1CC1 is critical for sensitising cells to ferroptosis. (A) *RB1* mRNA in control, and HepG2 cells with RB1CC1 KO or OV, treating with DMSO, RSL3 (1 μM) or erastin (10 μM) for 4 h. (B) ChIP experiments for detecting RB1CC1 binding within the *RB1* promoter in control and RB1CC1‐KO HepG2 cells treated with DMSO, RSL3 (1 μM) or erastin (10 μM) for 4 h. **p < .01 indicates significance between RSL3 and DMSO, or between erastin and DSMO. (C) Nuclear localisation of RB1CC1 in HepG2 cells following treating with RSL3 (1 μM) or erastin (10 μM) for indicated time. Scale bar, 10 μM. (D) Cellular fractionation experiments in HepG2 cells treated with RSL3 (1 μM) or erastin (10 μM) for indicated hours. (E) Subcellular localisation of truncated versions of RB1CC1 in HepG2 cells treated with DMSO, RSL3 (1 μM) or erastin (10 μM) for 4 h. Scale bar, 10 μm. (F) Phosphorylated and unphosphorylated RB1CC1 in HepG2 cells treated with DMSO, RSL3 (1 μM) or erastin (10 μM) for 4 h, as visualised by electrophoresis in gels containing Phostag™ reagent. (G) Schematic presentation of potential phosphorylation sites within the 499th–548th amino acid region of the RB1CC1 protein. (H) Phosphorylation of exogenous WT‐ or mutated‐RB1CC1 constructs, as indicated in HepG2 cells treated with DMSO, RSL3 (1 μM) or erastin (10 μM) for 4 h, as measured by gels containing Phostag™ reagent. Constructs expressing GFP was also co‐transfected as a loading control. (I) Subcellular localisation of WT‐, S537A‐ and S537E‐RB1CC1 constructs in HepG2 cells treated with DMSO, RSL3 (1 μM) or erastin (10 μM) for 4 h. Scale bar, 10 μm. (J) Cell death was measured in RB1CC1‐KO HepG2 cells reconstituted with WT‐, S537A‐, and S537E‐, RΔ5‐, RΔ6‐RB1CC1 constructs following treating with DMSO, RSL3 (1 μM), or erastin (10 μM) for 12 h. Statistical analysis was performed by Student's *t*‐test (B) or one‐way ANOVA (A and J). Data are presented as means ± SD from indicated samples. ***p < .001, **p < .01, *p < .05, indicates statistical significance and N.S. indicates non‐significance

RB1CC1 was first identified as a transcription factor that promotes *RB Transcriptional Corepressor 1* (*RB1*) gene transcription.^20^, ^21^ We found that *RB1* mRNA expression was also stimulated by RSL3 and erastin, which could be mitigated or reinforced by KO or OV of RB1CC1 (Figure 2A), suggesting that RB1CC1 may act as a transcription factor that enhances the transcription of target genes upon trigger of ferroptosis. To examine this possibility, ChIP experiments were performed. We found that RB1CC1 was recruited to the RB1CC1‐binding region (RBR) in the *RB1* promoter in control HepG2 cells treated with RSL3 and erastin but not in RB1CC1‐KO HepG2 cells. RB1CC1 did not bind a negative control region (NCR) in control cells (Figure 2B).

Transcription factors exert their functions largely in the nucleus.^29^ Therefore, we examined the localisation of RB1CC1 after the induction of ferroptosis. We discovered that RB1CC1 translocated from the cytoplasm into the nucleus at 1 h following triggering of ferroptosis and became obvious 4 h post‐treatment in HepG2 cells (Figure 2C). Cellular fractionation experiments in HepG2 cells further demonstrated ferroptosis‐inducible nuclear translocation of RB1CC1 (Figure 2D). We also confirmed ferroptosis‐inducible nuclear translocation of RBICC1 in LUAD H1299 and PACA SW1990 cells, indicating this may be likely a consistent mechanism across tumour cells (Figure S2A,B).

We next considered whether ferroptosis sensitivity was associated with the nuclear localisation of RB1CC1. We noticed that LUAD Calu‐1 cells were more sensitive to RSL3 and erastin as compared with LUAD H460 cells (Figure S2C). Unlike the weak cytoplasmic localisation of RB1CC1 in H460 cells, a stronger nuclear localisation of RB1CC1 was detected in Calu‐1 cells (Figure S2D). These results suggested that nuclear translocation of RB1CC1 is linked to ferroptosis.

Because cell density and cell adhesion have been shown to affect the erastin‐induced ferroptosis,^30^, ^31^ we examined the impact of differential cell density on RB1CC1 nuclear localisation. However, the intracellular localisation of RB1CC1 was not changed in response to changes of cell density (Figure S2E). These results also suggest that nuclear translocation of RB1CC1‐induced sensitisation of ferroptosis might be indispensable of cell density.

Apoptosis and ferroptosis share similar characteristics, and apoptosis can be stimulated upon induction of ferroptosis.^32^, ^33^ We next investigated whether induction of apoptosis can also stimulate nuclear translocation of RB1CC1. HepG2 cells were treated with cisplatin, a well‐established apoptosis inducer. Cisplatin had little effect on stimulating RB1CC1 nuclear translocation (Figure S2F); however, a significant induction of caspase 3/7 activities was observed (Figure S2G). These results suggest that RB1CC1 nuclear translocation might be one of the unique features of ferroptosis that do not overlap with apoptosis.

We next investigated the mechanisms underlying the nuclear translocation of RB1CC1 by constructing a panel of truncated RB1CC1 variants (Figure S2H). We found that RB1CC1 nuclear translocation was abolished upon deletion of amino acids 1–597 (RΔ3 vs. RWT, RΔ1, RΔ2 and RΔ4, Figure S2I). More precise analysis (RΔ5 vs. RΔ6) revealed that the 499–548 amino acid region in RB1CC1 is critical for ferroptosis‐inducible RB1CC1 nuclear translocation (Figure 2E).

Studies have shown that post‐translational modifications such as phosphorylation can stimulate nuclear translocation of proteins.^34^ Therefore, we conducted IB using a gel containing Phostag™ reagent, which allows the visualisation of phosphorylated proteins.^35^ We observed a supershift band representing phosphorylated RB1CC1 in cells treated with RSL3 and erastin (Figure 2F), suggesting that RB1CC1 is phosphorylated in a ferroptosis‐dependent manner. We narrowed down the potential phosphorylation sites to three serine residues, S533, S537 and S545, and two tyrosine residues, Y513 and Y528, within the 499–548 amino acid region (Figure 2G). We replaced these serine and tyrosine by alanine (A) to prevent phosphorylation or glutamic acid (E) to resemble hyperphosphorylation. Only the S537A mutation abolished the supershift of RB1CC1 following RSL3 and erastin treatment. By contrast, even in basal conditions, the S537E mutant resulted in a strong supershift of RB1CC1 (Figure 2H), indicating that RB1CC1 is phosphorylated at the S537 residue. Similarly, ferroptosis‐inducible nuclear translocation of RB1CC1 was linked to the phosphorylation at S537 residue (Figure 2I).

To examine whether phosphorylation of S537 influences the sensitivity of tumour cells to ferroptosis, RB1CC1‐KO HepG2 cells were reconstituted with RB1CC1^S537A^, RB1CC1^S537E^, RB1CC1^RΔ5^ and RB1CC1^RΔ6^. As shown in Figures 2J and S2J, ferroptosis sensitivity and generation of lipid ROS were reduced in RB1CC1^S537A^‐expressing HepG2 cells and induced in RB1CC1^S537E^‐expressing HepG2 cells compared with that in WT. In addition, compared with RB1CC1^RΔ5^, reconstitution with RB1CC1^RΔ6^ failed to restore sensitisation of HepG2 cells to ferroptosis and lipid ROS generation induced by RSL3 and erastin (Figures 2J and S2J). These results demonstrated that the 499–548 amino acid region that contains the potential S537 phosphorylation residue responsible for RB1CC1 nuclear translocation is critical for ferroptosis sensitisation in tumour cells.

To confirm the ferroptosis‐inducible S537 phosphorylation site, MS was performed. However, the S537 site was not identified in our MS data (Table S5). We speculate that this might be because of the low abundance of phosphorylation at this site.

### 3.3 A new role of RB1CC1 involving H4K12Ac histone modification in stimulating enhancer‐dependent transcription

**FIGURE 3:**
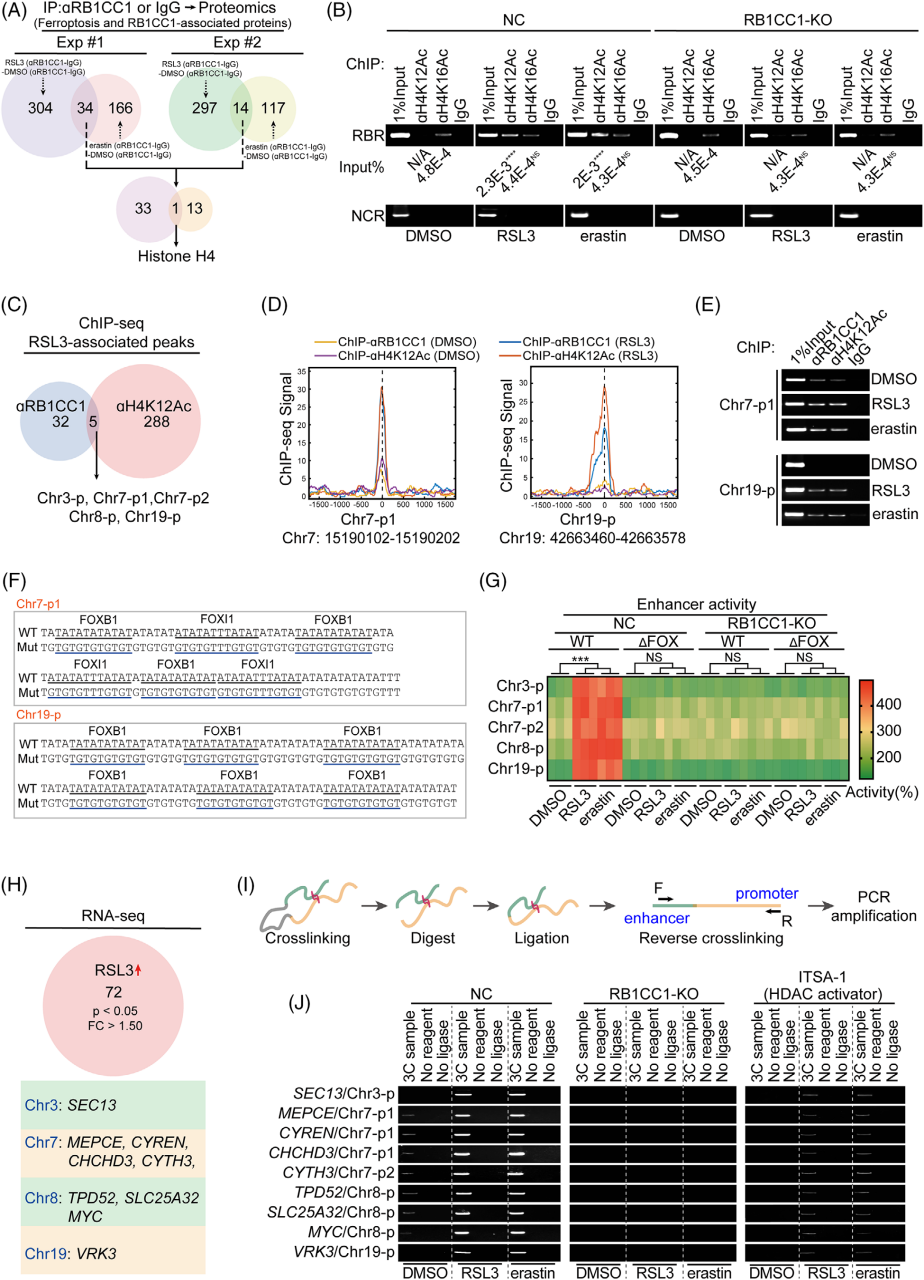
RB1CC1 links with H4K12Ac to mediate ferroptosis‐associated and enhancer‐dependent transcription. (A) Venn diagram showing the protein(s) interacted with RB1CC1 following RSL3 (1 μM) or erastin (10 μM) treatment for 4 h in experiment (Exp) #1 and #2. The proteins immunoprecipitated by anti‐RB1CC1 antibodies were subjected into mass spectroscopy (MS) for further analysis. (B) ChIP experiments for detecting enrichments within the *RB1* promoter using anti‐H4K12Ac, anti‐H14K16Ac or control IgG antibodies in control and RB1CC1‐KO HepG2 cells treated with DMSO, RSL3 (1 μM) or erastin (10 μM) for 4 h. ****p < .0001, indicates significance between RSL3 and DMSO, or between erastin and DMSO. N/A indicates not available. (C) Venn diagram showing peaks that revealed to be overlapped in ChIP‐Seq using anti‐RB1CC1 and anti‐H4K12Ac antibodies in HepG2 cells treated with RSL3 (1 μM) for 4 h. (D) The presentation of peaks for Chr7‐p1 and Chr19‐p from ChIP‐Seq experiments in HepG2 cells treated with DMSO or RSL3 (1 μM) for 4 h. (E) Verification of RB1CC1 binding and H4K12Ac histone modification within Ch7‐p1 and Chr19‐p in HepG2 cells treated with DMSO, RSL3 (1 μM) or erastin (10 μM) for 4 h. (F) DNA sequences of Chr7‐p1 and Chr19‐p with WT or mutated forkhead box (FOX)‐binding motifs, as indicated. (G) Heatmap showing the enhancer activity from indicated peaks with or without FOX‐binding motif in control and RB1CC1‐KO HepG2 cells treated with DMSO, RSL3 (1 μM) or erastin (10 μM) for 4 h. (H) RNA‐seq revealed genes that upregulated by RSL3 and located within the same chromosomes include the peaks that reveled by ChIP‐Seq in panel C. (I) Schematic representation of workflow for the chromosome conformation capture (3C) experiments. (J) Promoter–enhancer associations, as revealed by 3C experiment in control and HepG2 cells with RB1CC1 KO or pretreated with inhibitor‐1 of trichostatin A (ITSA‐1) (50 μM, 2 h), in the presence or absence of DMSO, RSL3 (1 μM) or erastin (10 μM) for 4 h. Statistical analysis was performed by Student's *t*‐test (B) or one‐way ANOVA (G). Data are presented as means ± SD from indicated samples. ****p < .0001, ***p < .001, indicates statistical significance and N.S. indicates non‐significance

Our results revealed that RB1CC1 is a ferroptosis‐associated transcription factor (Figure 2). To explore the mechanism underlying its transcriptional activity following trigger of ferroptosis, we performed immunoprecipitation (IP) followed by proteomics. The results identified 34 and 14 proteins were repeatedly identified as RSL3‐ and erastin‐inducible RB1CC1‐associated proteins in experiments #1 and #2, respectively. Notably, histone H4 was identified in both experiments #1 and #2 (Figure 3A and Table S6). Histone acetylation is closely associated with transcriptional reprogramming.^36^ Therefore, we evaluated H4K12Ac and H4K16Ac, two major histone H4 modifications, around the RBR within the *RB1* promoter. We found that H4K12Ac level was elevated in a ferroptosis‐ and RB1CC1‐dependent manner (Figure 3B), while no changes were observed in H4K16Ac levels. These results suggest that RB1CC1 may be involved in ferroptosis by increasing H4K12Ac histone modification levels.

To examine the ferroptosis‐ and RB1CC1‐dependent transcriptional program in tumour cells, ChIP‐Seq was performed using anti‐RB1CC1 and anti‐H4K12Ac antibodies in HepG2 cells. Five novel peaks were occupied with RB1CC1 and accompanied with remarkably increased H4K12Ac histone modification following RSL3 treatment in HepG2 cells (Figure 3C and Table S7). As shown in Figures 3D and S3A, the RB1CC1 and H4K12Ac peaks overlapped and sharp shaped. The enrichments of RB1CC1 and H4K12Ac within these five peaks were also experimentally verified to be associated with RSL3 and erastin (Figures 3E and S3B). De novo motif analysis revealed that all five peaks contained FOX‐family‐binding sites, but no significant RB1CC1‐binding motif was revealed (Figures 3F and S3C,D). This might be because the evidence for RB1CC1 as a transcription factor is still lacking. These peaks are far from the nearest ferroptosis‐associated genes (Figures 3H and S3E and Table S8), and therefore we reasoned that they might be distal enhancers.

To explore this possibility, the peaks were cloned into the pGL3 promoter plasmids. Luciferase activities from these plasmids were significantly induced following treatment with RSL3 and erastin (Figure 3G), suggesting that these peaks represent ferroptosis‐inducible enhancers. Ferroptosis‐induced elevation of enhancer activities was abolished upon deletion of all FOX motifs or upon RB1CC1 KO (Figure 3G), further indicating that ferroptosis‐inducible enhancer activity may rely on RB1CC1 interacting with FOX motifs. We then investigated whether FOX members are essential for RB1CC1 function. The enhancer activities from Chr7‐p1 and Chr19‐p were not altered following OV of FOXI1 and FOXB1 (Figure S3F). Moreover, co‐occupancies of RB1CC1 and FOXI1 were not detected at Chr7‐p1 or RBR (Figure S3G). These data indicated that RB1CC1 might merely share the FOX motif to stimulate ferroptosis‐inducible enhancer activity.

The classic model of eukaryotic gene expression involves the direct contact between a distal enhancer and a proximal promoter.^37^ To investigate whether RB1CC1‐associated enhancers play similar roles under ferroptotic conditions, potential ferroptosis‐associated genes were identified by RNA‐seq in cells without or with RSL3 treatment. RB1CC1‐associated enhancers were identified at Chr3, ‐7, ‐8 and ‐19 (Figure 3C). We therefore focused on RSL3‐upregulated genes within these chromosomes. As shown in Figure 3H, RNA‐seq predicted Chr3‐located *SEC3*, Chr7‐located *CYTH3*, *MEPCE*, *CYREN*, and *CHCHD3*, Chr8‐located *TPD52*, *SLC25A32*, and *MYC* and Chr19‐located *VRK3* as potential ferroptosis‐associated genes. Using 3C experiments to pinpoint interactions between RB1CC1‐associated enhancers and promoters of ferroptosis‐associated genes, we found that the interactions between them were remarkably strengthened when cells were treated with RSL3 and erastin. The interactions were abolished upon RB1CC1 KO and the treatment of ITSA‐1, a pan‐histone deacetylase activator (Figure 3I,J). Alterations of mRNA expression of these ferroptosis‐associated genes were also compromised (Figure S3H). Together, these data reveal a novel association between RB1CC1 and H4K12Ac histone modification in stimulating enhancer‐dependent transcription of ferroptosis‐associated genes.

### 3.4 RB1CC1 modulates mitochondria and ferroptosis via its target genes

**FIGURE 4:**
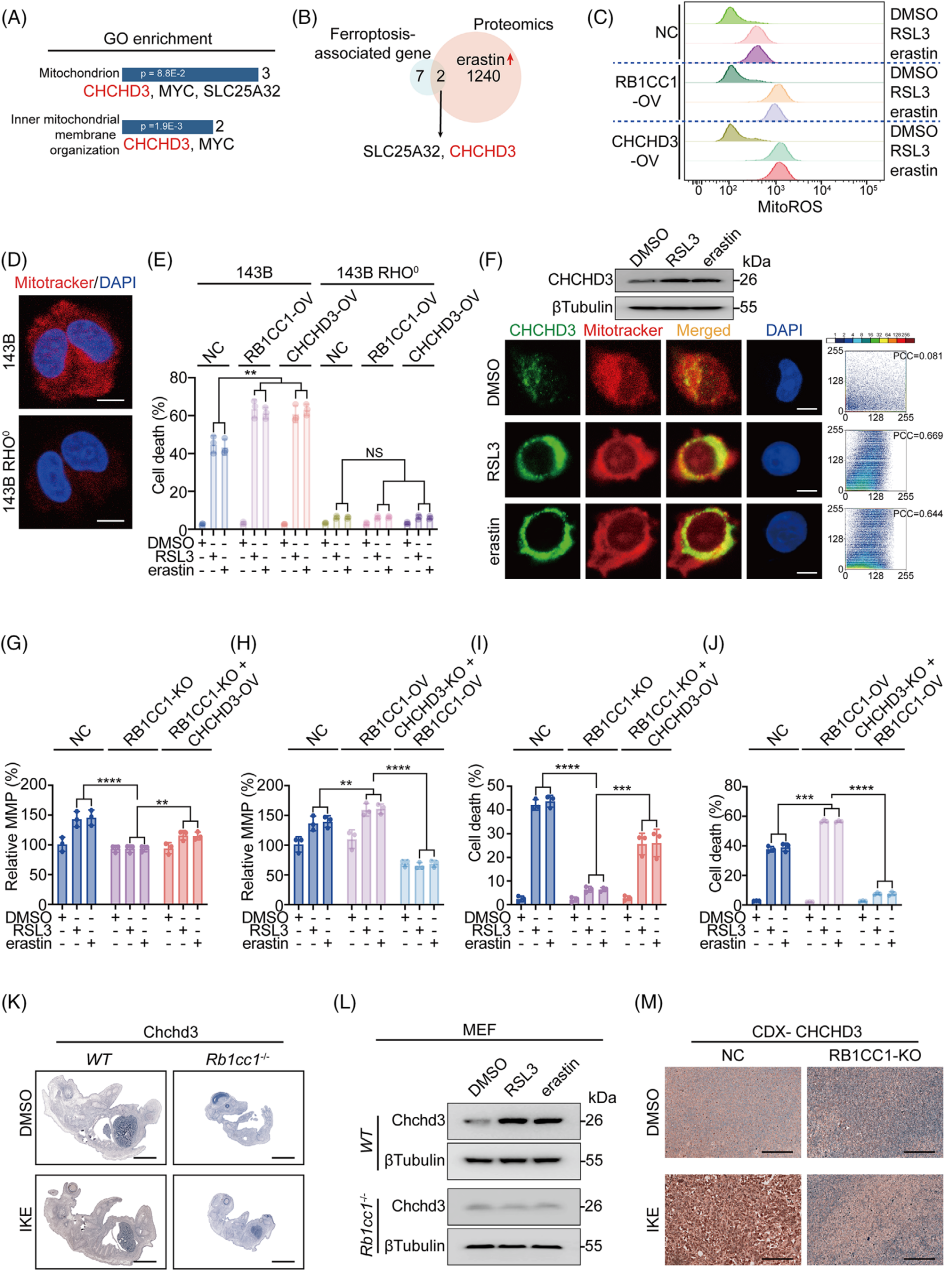
RB1CC1 target genes are associated with mitochondrion. (A) Gene ontology (GO) enrichment analysis identified mitochondrion is associated with ferroptosis‐associated genes. (B) Venn diagram showing the common candidates overlapped with ferroptosis‐associated genes and proteins that upregulated by erastin (10 μM) in HepG2 cells. (C) Smoothed histogram showing changes of mitoROS in control, HepG2 cells with RB1CC1 or CHCHD3 OV, and treated with DMSO, RSL3 (1 μM) or erastin (10 μM) for 16 h. (D) The presence of mitochondrion in 143B and 143B RHO° cells, as marked by mitotracker. Scale bar, 10 μm. (E) Cell death in 143B and 143B RHO° cells with or without RB1CC1 or CHCHD3 OV, in the presence of absence of DMSO, RSL3 (1 μM) or erastin (10 μM) for 12 h. (F) Western blotting of CHCHD3 protein expression in HepG2 cells treated with DMSO, RSL3 (1 μM) or erastin (10 μM) for 4 h. Co‐localisation of CHCHD3 and mitochondrion, as measured by IF using anti‐CHCHD3 antibodies and mitotracker. Co‐localisation between CHCHD3 and mitotracker was quantified using ImageJ software. On the co‐localisation scatter plot, the closer the scatter plot is to the diagonal line, the higher the degree of co‐localisation. Pearson's correlation coefficient (PCC) for fluorescent co‐localisation between CHCHD3 and mitotracker were obtained using ScatterJ analysis. PCC values from 0.5 to 1 indicate positive co‐localisation, and PCC values less than 0.5 indicates little co‐localisation. Scale bar, 10 μm. (G and I) MMP (G) and cell death (I) in control and HepG2 cells with RB1CC1 KO in the presence of absence of CHCHD3 OV, under the treatment of DMSO, RSL3 (1 μM) or erastin (10 μM) for 0.5 h (G) or 12 h (I). (H and J) The effects of RB1CC1 OV on MMP (H) and cell death (J) in control and HepG2 cells with CHCHD3 KO, under the treatment of DMSO, RSL3 (1 μM) or erastin (10 μM) for 0.5 h (H) or 12 h (J). (K) Chchd3 expression in *WT* and *Rb1cc1^–/–^* mouse foetuses obtained from cesarean section at E19 following pre‐administration with IKE (50 mg/kg) for 2 days in pregnant female mice. (L) Chchd3 in *WT* and *Rb1cc1^–/–^* MEF cells treated with DMSO, RSL3 (1 μM) or erastin (10 μM) for 4 h. (M) CHCHD3 in cell‐derived xenograft (CDX) that generated from Bel‐7402 cells with or without RB1CC1 KO following administrating mice with DMSO or IKE (50 mg/kg), as measured by immunohistochemistry (IHC). Scale bar, 200 μm. Statistical analysis was performed using one‐way ANOVA (E, G–J). Data are presented as means ± SD from indicated samples. ****p < .0001, ***p < .001, **p < .01, indicates statistical significance and N.S. indicates non‐significance

Our results show that enhancer binding is essential for RB1CC1 to stimulate the expression of ferroptosis‐associated genes (Figure 3). We next explored how the genes convey the roles of RB1CC1 in sensitising cells to ferroptosis. In GO enrichment analysis, three of the nine ferroptosis‐associated genes (Figure 3H) were predicted to be associated with mitochondria, and two of these were predicted to be involved in inner mitochondrial membrane organisation (Figure 4A). This suggests that RB1CC1 may sensitise ferroptosis via its target genes in a mitochondria‐dependent manner. Notably, CHCHD3 was the common gene involved (Figure 4A). CHCHD3 was also predicted to be upregulated by erastin via proteomics (Figure 4B). Thus, CHCHD3 was chosen for further study. On the basis of reports that mitoROS might be essential for ferroptosis,^38^, ^39^, ^40^ and our discovery that elevated mitoROS triggered by RSL3 and erastin was strengthened by overexpressing either RB1CC1 or CHCHD3 (Figure 4C), we speculated that RB1CC1 sensitises ferroptosis via targets such as CHCHD3 to increase mitoROS.

Our findings indicated that the RB1CC1–CHCHD3 axis is associated with mitoROS (Figure 4C). Whether mitochondria are involved in ferroptosis is so far still controversial.^38^, ^39^, ^40^ To address this question, we used osteosarcoma 143B RHO° cells (cells lacking mitochondrial DNA) and parental 143B cells (Figure 4D and^41^). Similar to overexpressing RB1CC1 conditions, overexpressing CHCHD3 also sensitised 143B cells to ferroptosis, but such effects were diminished in 143B RHO° cells (Figure 4E), suggesting that mitochondria are at least essential for RB1CC1 and CHCHD3 to sensitise ferroptosis. Moreover, CHCHD3 expression and CHCHD3 interaction with mitochondria were increased following RSL3 and erastin treatment (Figure 4F), further implying the critical roles of mitochondria in RB1CC1‐regulated ferroptosis. The MMP (Δψm) reflects mitochondria functional status.^42^ Dynamic monitoring of RSL3‐ and erastin‐treated HepG2 cells revealed that MMP was elevated very early and reached the highest value at 30 min following treatment; it declined to a lower‐than‐basal level at 4 h, and overexpressing RB1CC1 and CHCHD3 reinforced the overall extent of such effects (Figure S4A). However, instead of ‘parabola’‐like changes of MMP, treatment with RSL3 and erastin caused a consistent and gradual increase of mitoROS, which was reinforced by RB1CC1 and CHCHD3 (Figure S4B). Increased mitochondrial activity is accompanied with increased mitoROS formation, which might in turn facilitate a secondary release of mitoROS by disrupting mitochondrial function.^39^, ^43^ CHCHD3 is an inner MMP protein that maintains crista integrity and mitochondrial function.^44^ Therefore, we hypothesise that the consistent increasing of mitoROS following the trigger of ferroptosis is possibly initiated by increased mitochondrial function in a RB1CC1‐ and CHCHD3‐dependent fashion and is sustained by the subsequent ROS‐induced disruption of mitochondria.

To further verify the close RB1CC1–CHCHD3 relationship in modulating mitochondria and ferroptosis, MMP was examined at 0.5 h following RSL3 and erastin treatment. MMP was reduced following treatment with CCCP, an inhibitor of the mitochondrial electron transport chain (Figure S4C), thus verifying our evaluation system. RSL3 and erastin treatment did not significantly elevate MMP in RB1CC1‐KO HepG2 cells compared with the induction in control cells, but the effects were partially rescued by reconstituting CHCHD3 (Figures 4G and S4D). Moreover, RB1CC1 was ineffective in stimulating MMP when CHCHD3 was knocked out (Figures 4H and S4D). Similar outcomes were observed with ferroptosis‐associated cell death and lipid ROS generation (Figures 4I,J and S4E,F). In vivo experiments further demonstrated no IKE‐induced Chchd3 expression in to *Rb1cc1* ^–/–^ mouse foetuses compared with *WT* foetuses, indicating that IKE‐induced Chchd3 expression is Rb1cc1‐dependent (Figure 4K). In addition, in MEFs, Rb1cc1 was indispensable for RSL3 and erastin‐induced upregulation of Chchd3 (Figure 4L). Moreover, CHCHD3 in CDX was not induced by IKE when RB1CC1 was knocked out (Figure 4M). These results strongly suggest that CHCHD3 acts as a downstream effector of RB1CC1 that determines mitochondrial function and ferroptosis.

### 3.5 ELP3 interacts with RB1CC1 to modulate H4K12Ac histone modification and the ferroptosis‐associated transcription program

**FIGURE 5:**
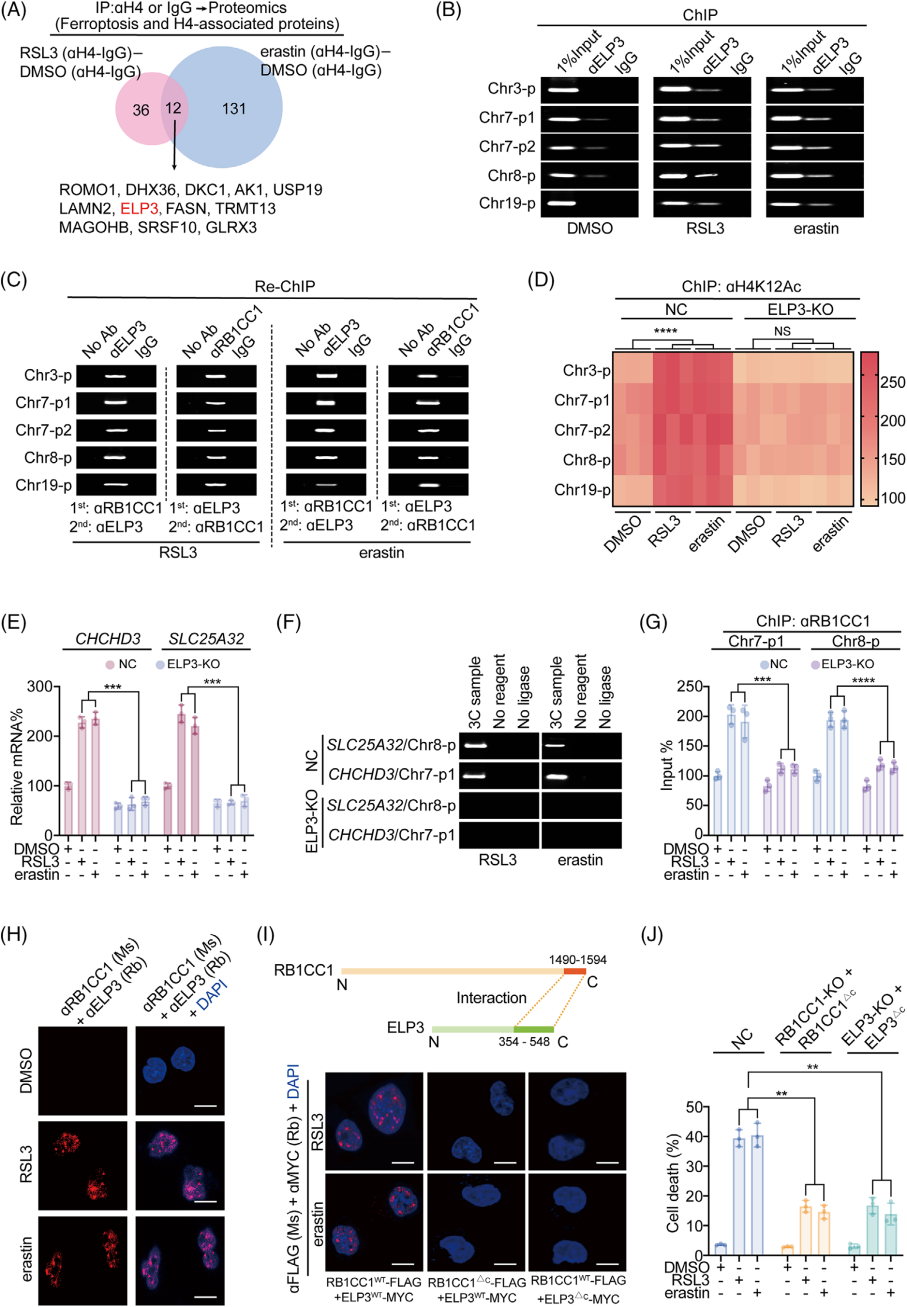
ELP3 is essential for RB1CC1‐mediated ferroptosis‐associated transcriptional reprogramming. (A) Venn diagram showing the common H4‐associated proteins following treating with DMSO, RSL3 (1 μM) and erastin (10 μM) for 4 h in HepG2 cells, as measured by IP using anti‐H4 or IgG antibodies followed by proteomics. (B) ChIP experiments showing ELP3 enrichment at ferroptosis‐associated peaks following treating with DMSO, RSL3 (1 μM) or erastin (10 μM) for 4 h in HepG2 cells. The IgG was parallel used as a negative control. (C) Re‐ChIP experiments showing co‐occupancy of RB1CC1 and ELP3 within the ferroptosis‐associated peaks in HepG2 cells treating with RSL3 (1 μM) or erastin (10 μM) for 4 h. (D) Heatmap showing changes of H4K12Ac within ferroptosis‐associated peaks in control and ELP3‐KO HepG2 cells treated with DMSO, RSL3 (1 μM) or erastin (10 μM) for 4 h. (E) *CHCHD3* and *SLC25A32* mRNA expression in control and ELP3‐KO HepG2 cells treated with DMSO, RSL3 (1 μM) or erastin (10 μM) for 4 h. (F) Chromosome conformation capture (3C) experiments demonstrating changes of promoter–enhancer associations in control and ELP3‐KO HepG2 cells treated with RSL3 (1 μM) or erastin (10 μM) for 4 h. (G) ChIP evaluating RB1CC1 enrichment within the Chr7‐p1 and Chr8‐p in control and ELP3‐KO HepG2 cells treated with DMSO, RSL3 (1 μM) or erastin (10 μM) for 4 h. (H) Interaction between RB1CC1 and ELP3 using proximity ligation assay (PLA) in HepG2 cells treated with DMSO, RSL3 (1 μM) or erastin (10 μM) for 4 h. (I) RB1CC1–ELP3 interaction relied on their C‐terminus, as measured by PLA in HepG2 cells expressing exogenous RB1CC1‐FLAG and ELP3‐Myc, as indicated, under the treatment of RSL3 (1 μM) or erastin (10 μM) for 4 h. (J) Cell death in control cells and RB1CC1‐KO and ELP3‐KO HepG2 cells reconstituted with exogenous RB1CC1^△C^‐FLAG and ELP3^△C^‐Myc following treating with DMSO, RSL3 (1 μM) or erastin (10 μM) for 12 h. Statistical analysis was performed using one‐way ANOVA (D, E, G, J). Data are presented as means ± SD from indicated samples. ****p < .0001, ***p < .001, **p < .01, indicates statistical significance and N.S. indicates non‐significance

Our results showed that H4K12Ac histone modification is essential for RB1CC1 to regulate ferroptosis‐associated transcriptional reprogramming (Figure 3). To the best of our knowledge, no studies have examined whether RB1CC1 regulates histone modification. To seek RB1CC1‐associated histone regulators, we immunoprecipitated H4 using anti‐H4 antibodies and conducted proteomics experiments. The results identified 48 RSL3‐induced H4‐associated proteins and 143 erastin‐induced H4‐associated proteins, with 12 proteins overlapping between the groups (Figure 5A and Table S9). STRING database was used to reveal protein–protein associations; only ELP3 was previously reported as a histone acetyltransferase (HAT, Figure S5A and^45^). ChIP and Re‐ChIP experiments demonstrated co‐occupancy of EPL3 and RB1CC1 within the ferroptosis‐associated peaks in cells treated with RSL3 and erastin (Figure 5B,C). Moreover, RSL3‐ and erastin‐induced elevation of H4K12Ac within ferroptosis‐associated peaks was ELP3 dependent (Figure 5D). We evaluated the expressions of *CHCHD3* and *SLC25A32*, two mitochondria‐associated targets of RB1CC1 (Figures 3H and 4A), and found that induction of the two genes following treatment with RSL3 and erastin occurred via ELP3 (Figure 5E). We next investigated whether ELP3 was essential for RB1CC1‐mediated transcriptional reprogramming. RB1CC1 is critical for enhancer–promoter associations (Figure 3J), and 3C experiments were performed to evaluate whether ELP3 affects spatial transcription upon trigger of ferroptosis. We found that ELP3 was at least indispensable for Chr8‐p‐*SLC25A32* promoter and Chr7‐p1‐*CHCHD3* promoter associations (Figure 5F). Furthermore, RSL3‐ and erastin‐induced RB1CC1 binding with the two enhancers were abolished when ELP3 was knocked out (Figure 5G). These results indicated that ELP3 is essential for RB1CC1 to stimulate ferroptosis‐associated transcriptional reprogramming.

Next, we investigated the molecular basis for the potential RB1CC1–ELP3 interaction. PLA was first performed, and the protein‐protein interactions (PPI) were only detected when both anti‐RB1CC1 and anti‐ELP3 antibodies were added in HepG2 cells following RSL3 and erastin treatment (Figures 5H and S5B).These results suggest that RB1CC1 and ELP3 are in close proximity upon trigger of ferroptosis. Previous studies demonstrated that the C‐terminus regions of RB1CC1 and ELP3 function as PPI domains.^46^, ^47^, ^48^ Consistent with this finding, we found that the RB1CC1 and ELP3 PPI were dependent on the C‐terminus (Figure 5I). We then evaluated whether the C‐terminus regions of RB1CC1 and ELP3 are essential for sensitising cells to ferroptosis by generating a panel of mutant constructs lacking the C‐terminus (RB1CC1^ΔC^ and ELP3^ΔC^). To avoid potential competitive effects by the mutant constructs, we used RB1CC1‐KO and ELP3‐KO HepG2 cells and reconstituted cells with the mutant constructs. Compared with the controls HepG2 cells, RB1CC1^ΔC^‐ and ELP3^ΔC^‐expressing HepG2 cells displayed less sensitivity to RSL3‐ and erastin‐induced cell death, mitoROS and lipid ROS generation (Figures 5J and S5C,D). Together, these findings indicate that the RB1CC1–ELP3 interaction is critical for sensitising tumour cells to ferroptosis.

### 3.6 Activating JNK stimulates RB1CC1 to sensitise ferroptosis and inhibit tumourigenesis

**FIGURE 6:**
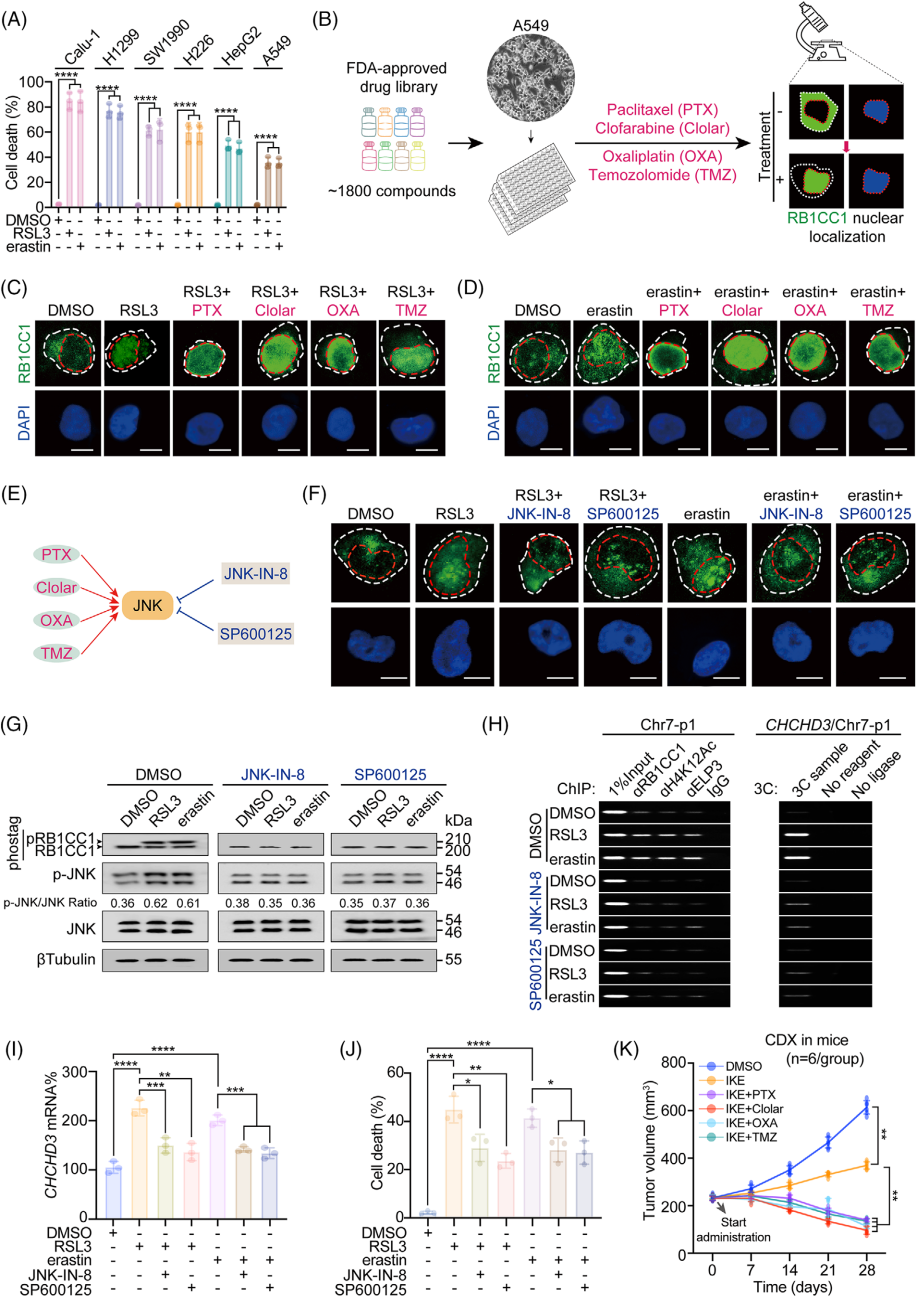
Activating JNK reinforces the role of RB1CC1 to sensitise ferroptosis. (A) Cell death in a serial of tumour cells, as indicated, following treating with DMSO, RSL3 (1 μM) or erastin (10 μM) for 12 h. (B) Workflow of identifying the potential drugs that might stimulate nuclear translocation of RB1CC1 in A549 cells. (C and D) Subcellular localisation of RB1CC1 in A549 cells treated with DMSO, RSL3 (1 μM) (C) or DMSO, erastin (10 μM) (D) for 4 h, in the presence or absence of paclitaxel (PTX) (50 pM), clofarabine (Clolar) (10 μM), oxaliplatin (OXA) (50 μM) or temozolomide (TMZ) (50 μM). Scale bar, 10 μm. (E) Schematic presentation of activators and inhibitors of JNK. (F) Subcellular localisation of RB1CC1 in A549 cells following treating with DMSO, RSL3 (1 μM) or erastin (10 μM) for 4 h, with or without JNK‐IN‐8 (1 μM) or SP600125 (10 μM). Scale bar, 10 μm. (G) Phosphorylation of RB1CC1 and JNK in A549 cells treated with DMSO, RSL3 (1 μM) or erastin (10 μM) for 4 h, with or without JNK‐IN‐8 (1 μM) or SP600125 (10 μM), as measured by gels containing with or without Phostag™. (H) ChIP (left panel) and chromosome conformation capture (3C) experiments (right panel) showing RB1CC1 and ELP3 binding, H4K12Ac histone modification at Chr7‐p1 and *CHCHD3* promoter‐Chr7‐p1 association in A549 cells treated with DMSO, RSL3 (1 μM) or erastin (10 μM) for 4 h, with or without JNK‐IN‐8 (1 μM) or SP600125 (10 μM). (I and J) *CHCHD3* mRNA (I) and cell death (J) in A549 cells treated with DMSO, RSL3 (1 μM) or erastin (10 μM), with or without JNK‐IN‐8 (1 μM) or SP600125 (10 μM) for 4 h (I) or 12 h (J). (K) Growth of xenografts cell‐derived xenograft (CDX) that generated by A549 cells treated with DMSO or IKE (50 mg/kg), in the presence or absence of PTX (20 mg/kg), Clolar (10 mg/kg), OXA (10 mg/kg) or TMZ (40 mg/kg). The tumour volumes were monitored at indicated time. *n* = 6 mice/group. Statistical analysis was performed using one‐way ANOVA (A) or Student's *t*‐test (I and J) or two‐way ANOVA (K). Data are presented as means ± SD from indicated samples. ****p < .0001, ***p < .001, **p < .01, *p < .05, indicates statistical significance

Our findings have shown that RB1CC1 sensitises tumour cells to ferroptosis (Figures 1, 2, 3, 4, 5). We therefore performed a search for drugs that reinforce the function of RB1CC1, with the aim of identifying potential cancer therapeutic drugs. After comparing the sensitivities of a series of tumour cell lines to ferroptosis, we noticed that the LUAD A549 cell line demonstrated the least sensitivity among the tumour cell lines tested (Figure 6A). Hence, A549 cells were selected as a relative ferroptosis‐resistant cell line to investigate the efficacy of potential drug(s).

Our findings indicated nuclear translocation is a hallmark for RB1CC1 to sensitise cells to ferroptosis. We thus treated A549 cells with an FDA‐approved drug library (Selleckchem) containing ∼1800 compounds and investigated the drugs that stimulated RB1CC1 nuclear translocation. Our analysis identified four drugs, PTX, Clolar, OXA and TMZ, that induced these effects (Figure 6B). In addition, the four drugs synergistically enhanced the efficacies of RSL3 and erastin to stimulate RB1CC1 nuclear translocation in A549 cells (Figure 6C,D). The four drugs also enhanced the effect of RSL3 and erastin to induce cell death, lipid ROS and mitoROS generation (Figure S6A–F).

JNK is a common target and is phosphorylated and activated by the four drugs (illustrated in Figure 6E and^49^, ^50^, ^51^, ^52^). We thus examined whether JNK is involved in the nuclear translocation of RB1CC1. We used JNK‐IN8 and SP600125, two well‐established JNK inhibitors, in cells treated with RSL3 and erastin. As shown in Figures 6F,G and S6G, phosphorylation of JNK and nuclear translocation of RB1CC1 was prevented after treatment with the JNK inhibitors. Our results showed that phosphorylation of RB1CC1 is essential for its nuclear translocation (Figure 2F–I), and we found that RSL3‐ and erastin‐induced phosphorylation of RB1CC1 was also prevented by JNK‐IN‐8 and SP600125 (Figure 6G). These results demonstrated that JNK activators are prone to reinforce the phosphorylation of RB1CC1 and its nuclear translocation.

Additionally, using ferroptosis‐resistant H460 lung cancer cells,^53^ we evaluated whether targeting the RB1CC1 pathway removes cellular resistance to ferroptosis. However, treating cells with JNK agonists Clolar and OXA neither stimulated nuclear translocation of RB1CC1 nor influenced ferroptosis sensitivity in H460 cells (Figure S6H,I), suggesting that JNK agonists might only reinforce ferroptosis‐based therapy in ferroptosis‐inducible tumour cells.

Next, we examined whether JNK influences ferroptosis‐associated transcriptional reprogramming. RSL3‐ and erastin‐induced enrichment of RB1CC1 and ELP3 and elevation of H4K12Ac within Chr7‐p1 were prevented in cells treated with JNK‐IN‐8 and SP600125 (Figure 6H). In such conditions, RSL3‐ and erastin‐induced Chr7‐p1‐*CHCHD3* enhancer–promoter association was also abolished (Figure 6H). Similar results were observed regarding *CHCHD3* and *RB1* mRNAs in A549 cells (Figures 6I and S6J). Furthermore, we proved that the Rb1cc1‐dependent induction of Chchd3 following treatment with RSL3 and erastin in MEF cells was dependent on JNK (Figure S6K). Inhibiting JNK mitigated RSL3‐ and erastin‐induced MMP, mitoROS generation and cell death (Figures 6J and S6L,M), further demonstrating the importance of JNK to modulate RB1CC1 in ferroptosis. Whether PTX, Clolar, OXA and TMZ strengthen the anti‐tumourigenic efficacy of IKE was investigated in CDX mouse models that were generated from A549 cells. Co‐administrating IKE with the above four compounds resulted in inhibition and even regression of tumour growth (Figure 6K). Together, these results indicate that activating JNK stimulates RB1CC1 to sensitise ferroptosis and inhibits tumour growth.

### 3.7 Clinical and translational significance of the present study

**FIGURE 7:**
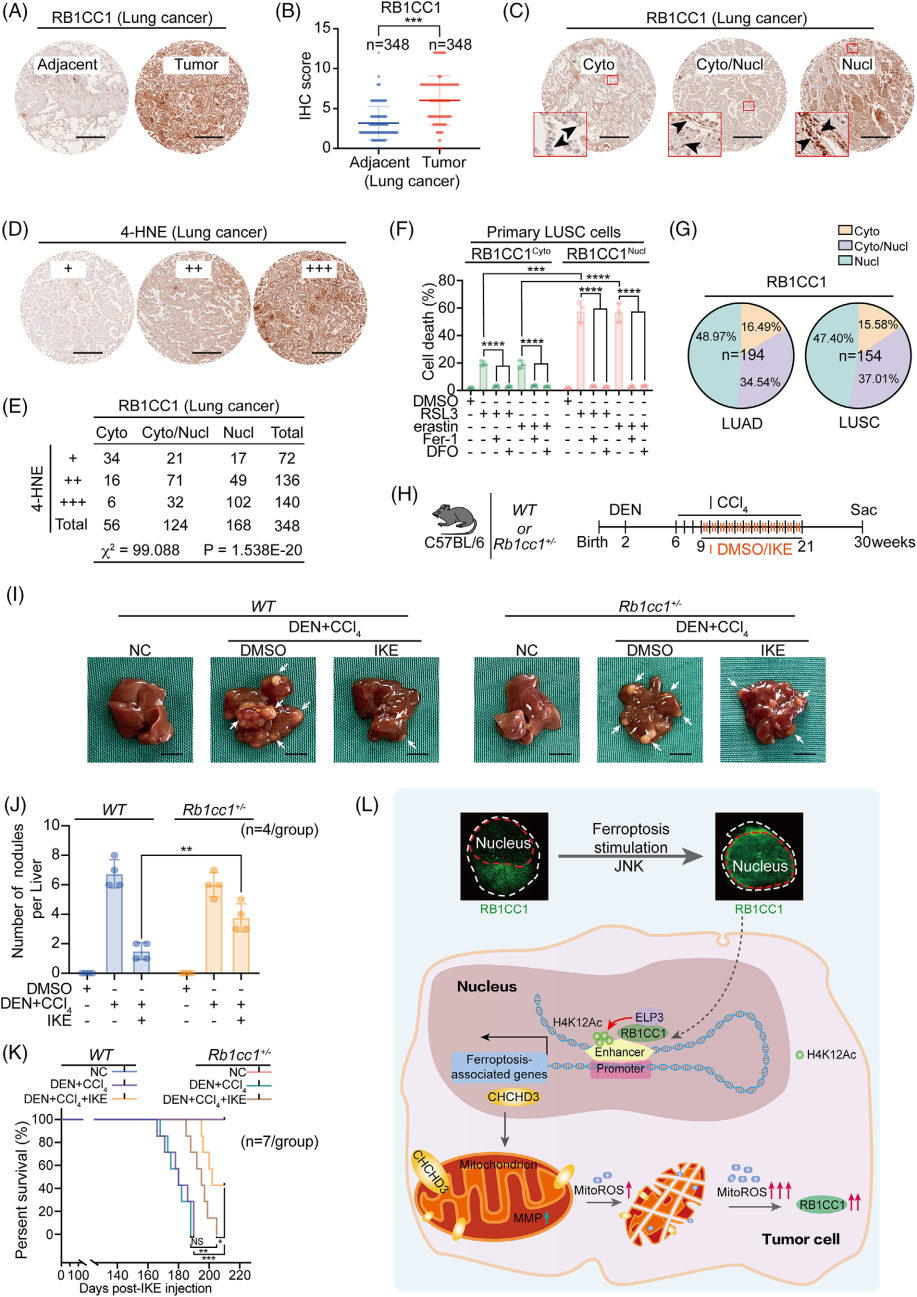
Clinical and translational significance of the study. (A) Representative tissue microarray (TMA) images of RB1CC1 in tumour and paired adjacent lung tissues. Scale bar, 100 μm. (B) Immunohistochemistry (IHC) scores for evaluating RB1CC1 in tumour and paired adjacent lung tissues from TMA. *n* = 348/group. (C) Representative TMA images for different subcellular localisation of RB1CC1 in lung cancer. Arrow heads indicate RB1CC1 subcellular localisation. (D) Representative IHC images for 4‐HNE in TMA that same as the ones in panel C. (E) The association between RB1CC1 and 4‐HNE in lung cancer, as evaluated by *χ* ^2^‐test. (F) Cell death in primary lung squamous cell carcinoma (LUSC) cells with distinct cytosol and nuclear subcellular localisation of RB1CC1 following treating with DMSO, RSL3 (1 μM) or erastin (10 μM), in the presence or absence of Fer‐1 (2 μM) or DFO (100 μM) for 12 h. (G) The percentage of different subcellular localisation of RB1CC1 in lung adenocarcinoma (LUAD) (*n* = 194) and LUSC (*n* = 154). (H) Workflow for mice treatment. (I) Macroscopic liver appearance of *WT* or *Rb1cc1^+/–^* mice treated with or without diethylnitrosamine (DEN) and carbon tetrachloride (CCl_4_) and administered with DMSO or IKE. *n* = 4 mice/group, scale bar, 10 mm. (J) Tumour burden in mice liver from panel I. (K) Survive curve for mice under the same treatment as that in panel I. (L) Schematic presentation of the present study. Briefly, nuclear translocation of RB1CC1 occurs upon induction of ferroptosis. Nuclear RB1CC1 recruits histone acetyltransferase ELP3, which facilitates H4K12Ac histone modification within enhancers. Such effects reinforce enhancer–promoter association and finally stimulate ferroptosis‐associated transcriptional reprogramming. CHCHD3 is such a target gene of RB1CC1. Trigger of ferroptosis stimulates RB1CC1 and CHCHD3‐dependent mitochondrion function at a very early stage, which is essential for initiating an increase of mitoROS). As a consequence, tumour cells to ferroptosis are sensitised. Statistical analysis was performed using Student's *t*‐test (B, F, J) or one‐way ANOVA (F), survival curves (K) were compared with the log‐rank test. Data are presented as means ± SD from indicated samples. ****p < .0001, ***p < .001, **p < .01, *p < .05, indicates statistical significance and N.S. indicates non‐significance

We next evaluated the clinical and translational significance of RB1CC1 in tumours. Analysis of the UALCAN database revealed that RB1CC1 was upregulated in HCC and LUSC (Figure S7A). We thus mainly focused on liver and lung cancers. RB1CC1 was also upregulated in our institutional lung cancer cohort (*n* = 348; Figure 7A,B and S7B). As increased ROS reflects high metabolic activity in tumour cells and ROS determines RB1CC1 expression (Figure 1A and^17^), the upregulation of RB1CC1 in lung cancer can be explained by the high malignant property. Our results showed that nuclear translocation of RB1CC1 sensitises ferroptosis (Figure 2). Therefore, we examined the subcellular localisation of RB1CC1 and its association with ferroptosis sensitivity in lung cancer. On the basis of RB1CC1 subcellular localisation, lung cancer was divided into cytoplasmic (cyto), nuclear (nucl) and joint cytoplasmic‐nuclear (cyto/nucl) subtypes (Figure 7C). We evaluated the levels of 4‐HNE in 348 lung cancer specimens and found that the cyto‐subtype had the lowest 4‐HNE levels, while the nucl‐subtype had the highest ones (Figure 7D,E), demonstrating that lipid peroxidation is possibly dependent on RB1CC1 subcellular localisation. The sensitivity of ferroptosis was tested in primary LUSC cells in which RB1CC1 was strictly localised in nucleus or the cytoplasm (Figure S7C). Primary LUSC cells with RB1CC1 nuclear localisation demonstrated increased sensitivity to ferroptosis as compared with cells with cytoplasmic localisation (Figure 7F). Approximately 48.97% (95/194) LUAD and 47.4% (73/154) LUSC specimens displayed RB1CC1 nuclear subcellular localisation (Figure 7G), suggesting a large portion of lung cancer may benefit from ferroptosis‐based therapy.

We next evaluated the importance of RB1CC1 in liver cancer using DEN‐ and CCl_4_‐induced HCC mouse models, as these are suitable for the study of liver cancer in vivo. As described in Figure 1H, *Rb1cc1* ^–/–^ mice die shortly after birth, but *Rb1cc1* ^+/–^ mice are born normally, having body weights and physical features indistinguishable from *WT* mice. Therefore, we generated in vivo liver cancer models in *WT* and *Rb1cc1* ^+/–^ mice. We found that the capacity of DEN/CCl_4_ to induce liver tumourigenesis was similar in *WT* and *Rb1cc1* ^+/–^ mice (Figure 7H,I). Nonetheless, the inhibitory effect of IKE on tumour growth was remarkably reduced in *Rb1cc1* ^+/–^ mice as compared with that in *WT* mice (Figure 7I,J). The survival outcomes were compromised (Figure 7K). Together, these findings indicate that Rb1cc1 is critical for the efficacy of ferroptosis‐based anti‐tumour treatments.

## 4 DISCUSSION

As a result of advances in multi‐omic screening technologies, the understanding of the regulatory mechanisms of ferroptosis in tumour cells has continued to improve. Emerging evidence has demonstrated that a variety of tumour‐related proteins and signalling pathways are involved in the regulation of ferroptosis.^10^, ^14^, ^24^ Shuttling of terminal effector(s) in or out of the nucleus is a hallmark of the activation or suppression of certain signalling pathways.^54^ Our in‐depth in vitro and in vivo findings identified RB1CC1 as one of the key regulators of ferroptosis‐associated signalling and revealed that nuclear translocation of RB1CC1 is critical for inducing transcriptional reprogramming and sensitising ferroptosis. We further found that this signalling stimulates mitochondria and facilitates mitoROS generation, and RB1CC1 is positively controlled by ROS and JNK. These findings thus establish the regulation and function of RB1CC1‐associated signalling to sensitise tumour cells to ferroptosis (Figure 7L).

Previous studies have demonstrated roles for RB1CC1 in regulating cell growth, differentiation, apoptosis and autophagy.^20^, ^21^ To the best of our knowledge, whether RB1CC1 influences ferroptosis had not been explored. After the identification of RB1CC1 as a transcription factor to stimulate transcription of the tumour suppressor *RB1*, several studies investigated its functions in tumourigenesis.^20^, ^21^ Other studies showed that RB1CC1 is critical for autophagy in tumour cells.^19^, ^23^ RB1CC1 forms a complex with Unc‐51‐like autophagy activating kinase 1 (ULK1), which acts as a downstream effector of mTOR; thus, RB1CC1 participates in mTOR‐regulated autophagy process.^55^ However, the pro‐autophagic activity of RB1CC1 is mitigated following its binding with p53.^56^ Later studies revealed that the distinct function of RB1CC1 depends on its subcellular localisation. For instance, RB1CC1 acts as a transcription factor in the nucleus, while it regulates autophagy in the cytoplasm.^22^, ^23^, ^55^ In the present study, we demonstrated that RB1CC1 is indeed a transcription factor that regulates ferroptosis‐associated transcription in the nucleus. Moreover, we found that RB1CC1 sensitises ferroptosis in an autophagy‐independent manner. Therefore, the cytoplasm‐nucleus shuttle of RB1CC1 might be essential for the switch of the distinct functions of RB1CC1 between autophagy and ferroptosis.

In the past few years, researchers have been paying increasing attention to the role of mitochondria in ferroptosis. The mitochondrial matrix and crest are the major sites for oxidative metabolism in eukaryotes.^38^ Mitochondria are rich in lipids and iron,^40^ and some studies proposed that mitochondria are an important locus for the occurrence of ferroptosis.^38^, ^40^ Although mitochondria are one of the major sources for ROS production, excessive accumulation of mitoROS can reversely increase mitochondrial membrane permeability and eventually impair mitochondrial function.^39^ Morphological changes of mitochondria, such as the disappearance of cristae and shrinkage of membrane, are regarded as characteristics of ferroptosis.^15^ Gao et al.^38^ reported that mitochondria are essential for ferroptosis induced by cysteine deprivation, which causes hyperpolarisation of MMP and accumulation of lipid peroxide. Our findings also demonstrated that RB1CC1‐mediated transcriptional reprogramming is critical to stimulate MMP hyperpolarisation and mitoROS production in the very early stage upon trigger of ferroptosis. We identified the CHCHD3 gene as a RB1CC1 target gene that is positively regulated by RB1CC1. As part of the mitochondrial inner membrane structure regulation complex, CHCHD3 plays an important role in improving the stability and cristae morphology of mitochondria, thereby providing a stable environment for the enhancement of mitochondrial function.^44^ These findings support that RB1CC1‐mediated upregulation of CHCHD3 stimulates mitochondrial function and increases ROS production to sensitise tumour cells to ferroptosis. Together these findings indicate that mitochondria are essential for ferroptosis.

The function of RB1CC1 to reinforce mitochondrial function relies on ELP3‐mediated H4K12Ac histone modification. Our findings demonstrated that the C‐terminuses of RB1CC1 and ELP3 were critical for their interaction and impact on ferroptosis. The C‐terminus of RB1CC1 contains a so‐called ‘claw’ domain because it is composed of five β sheets and a short α helix and is partly shaped like a curved finger.^46^ The ‘claw’ domain is important for forming heterodimers between RB1CC1 and other proteins, such as cell cycle progression 1 (CCPG1), nuclear dot protein 52 (NDP52), nak‐associated protein 1 (NAP1) and sequestosome 1 (SQSTM1).^47^, ^57^, ^58^ The C‐terminus is also essential for the function of ELP3, since the iconic‐free radical S‐adenosylmethionine (SAM) and HAT domains in the C‐terminal domain are present in all ELP3 proteins from archaea to humans.^45^ In addition to its histone acetylation function, the HAT domain of ELP3 also provides a function that is essential for transcriptional activation of target genes.^45^, ^48^ While our results identified that the RB1CC1–ELP3 interaction occurs within the C‐terminuses of both proteins, the exact sites for the regulation of ferroptosis‐associated transcriptional reprogramming have not been determined and require more in‐depth studies.

JNK belongs to the MAPK superfamily. JNK participates in the signal transduction related to various growth factors, cytokines, mitogens and hormone receptors after activation and is essential for cell proliferation, differentiation and apoptosis.^59^ Moreover, JNK signalling is also activated under oxidative stress.^60^ Notably, under certain conditions, ROS stimulates JNK.^61^ This also explains our findings that RB1CC1 is underlying controlled by both ROS and JNK once upon induction of ferroptosis. Upregulation and nuclear translocation of RB1CC1 occurs rapidly before ferroptosis‐associated cell death, hinting that ferroptosis can be manipulated by targeting RB1CC1. Several agonists and antagonists of JNK have been developed and some are FDA approved. Uncovering the pro‐ferroptotic function of JNK agonists is of great translational significance and may provide more choices to reinforce ferroptosis‐based therapy against tumours.

## 5 CONCLUSION

Our study identified RB1CC1 as a key regulator to sensitise ferroptosis in tumour cells and proposes a novel ferroptosis‐associated regulatory signalling pathway. We strongly recommend that targeting RB1CC1 is a promising perspective to reinforce ferroptosis‐based therapy against tumours.

## CONFLICT OF INTEREST

The authors declare no potential conflicts of interest.

## Supporting information

Supplementary Table 1.pdf

Supplementary Table 2.pdf

Supplementary Table 3.xlsx

Supplementary Table 4.xlsx

Supplementary Table 5.0.docx

Supplementary Table 6.0.xlsx

Supplementary Table 7.0.xlsx

Supplementary Table 8.0.xlsx

Supplementary Table 9.0.xlsx

2022‐0204Revision supplementary figures legends.docx
