# Supplementary material for: Tumour cells are sensitised to ferroptosis via RB1CC1‐mediated transcriptional reprogramming
Source: Clin Transl Med. 2022 Feb 27;12(2):e747. doi: 10.1002/ctm2.747 (PMC8882240; doi:10.1002/ctm2.747)
Supplement: Supplementary file 1 — Supplementary Table 1.pdf [file CTM2-12-e747-s008.pdf]

**Supplementary Table 1. SgRNAs and Primers in the study.**

sgRNA sequence

| Name | 5'-3' |
|---|---|
| RB1CC1-KO | GGCCGACTCTGACTGAGCCT |
| CHCHD3-KO | GATTGACACAGTGCATATAC |
| ELP3-KO | GGCACGGATAGCTCTCATGG |

Primers used for plasmid construction

| Name | 5'-3' |
|---|---|
| RB1CC1-OV-F | GCGCGAATTCATGAAGTTATATGTATTTCTGGTTA |
| RB1CC1-OV-R | GCGCGGTACCTTATTTCAGCATCTTCACCATGTAC |
| CHCHD3-OV-F | GCGCGAATTCATGGGTGGGACCACCAGCACCCGCC |
| CHCHD3-OV-R | GCGCGGTACCTTATCCTCCCTTCTCAAGCATGCTC |
| RWT-RB1CC1-FLAG-F | GCGCGGTACCATGAAGTTATATGTATTTCTGGTTA |
| RWT-RB1CC1-FLAG-R | GCGCGAATTCTCACTTATCGTCGTCATCCTTGTAATCTTATACTTTCTTATTCCATGATACG |
| R△1-RB1CC1-FLAG-F | GCGCGGTACCATGAAGTTATATGTATTTCTGGTTA |
| R△1-RB1CC1-FLAG-R | GCGCGAATTCTCACTTATCGTCGTCATCCTTGTAATCTTATACTTTCTTATTCCATGATACG |

| | |
|---|---|
| R△2-RB1CC1-FLAG-F | GCGCGGTACCATGAAGTTATATGTATTTCTGGTTA |
| R△2-RB1CC1-FLAG-R | GCGCGAATTCTCACTTATCGTCGTCATCCTTGTAATCTTATACTTTCTTATTCCATGATACG |
| R△3-RB1CC1-FLAG-F | GCGCGGTACCATGAAGTTATATGTATTTCTGGTTA |
| R△3-RB1CC1-FLAG-R | GCGCGAATTCTCACTTATCGTCGTCATCCTTGTAATCTTATACTTTCTTATTCCATGATACG |
| R△4-RB1CC1-FLAG-F | GCGCGGTACCATGAAGTTATATGTATTTCTGGTTA |
| R△4-RB1CC1-FLAG-R | GCGCGAATTCTCACTTATCGTCGTCATCCTTGTAATCTTATACTTTCTTATTCCATGATACG |
| R△5-RB1CC1-FLAG-F | GCGCGGTACCATGAAGTTATATGTATTTCTGGTTA |
| R△5-RB1CC1-FLAG-R | GCGCGAATTCTCACTTATCGTCGTCATCCTTGTAATCTTATACTTTCTTATTCCATGATACG |
| R△6-RB1CC1-FLAG-F | GCGCGGTACCATGAAGTTATATGTATTTCTGGTTA |
| R△6-RB1CC1-FLAG-R | GCGCGAATTCTCACTTATCGTCGTCATCCTTGTAATCTTATACTTTCTTATTCCATGATACG |
| Y513A-RB1CC1-FLAG-F1 | GCGCGGTACCATGAAGTTATATGTATTTCTGGTTA |
| Y513A-RB1CC1-FLAG-R1 | CCCTGGCGTGTTTTATGAAC |
| Y513A-RB1CC1-FLAG-F2 | GTTCATAAAACACGCCAGGG |
| Y513A-RB1CC1-FLAG-R2 | GCGCGGATCCATGGAGCAGAAACTCATCTCTGAAGAGGATCTGATGAAGTTATATGTATTTCTGGTTA |
| Y528A-RB1CC1-FLAG-F1 | GCGCGGTACCATGAAGTTATATGTATTTCTGGTTA |
| Y528A-RB1CC1-FLAG-R1 | TGCTTCAGCTAATCTCTTTC |
| Y528A-RB1CC1-FLAG-F2 | GAAAGAGATTAGCTGAAGCA |
| Y528A-RB1CC1-FLAG-R2 | GCGCGGATCCATGGAGCAGAAACTCATCTCTGAAGAGGATCTGATGAAGTTATATGTATTTCTGGTTA |
| S533A-RB1CC1-FLAG-F1 | GCGCGGTACCATGAAGTTATATGTATTTCTGGTTA |

| | |
|---|---|
| S533A-RB1CC1-FLAG-R1 | GATTCCCTTTTTGCTTTTTC |
| S533A-RB1CC1-FLAG-F2 | GAAAAAGCAAAAAGGGAATC |
| S533A-RB1CC1-FLAG-R2 | GCGCGGATCCATGGAGCAGAAACTCATCTCTGAAGAGGATCTGATGAAGTTATATGTATTTCTGGTTA |
| S537A-RB1CC1-FLAG-F1 | GCGCGGTACCATGAAGTTATATGTATTTCTGGTTA |
| S537A-RB1CC1-FLAG-R1 | AATTTCCCAAAGGCTTCCCT |
| S537A-RB1CC1-FLAG-F2 | AGGGAAGCCTTTGGGAAATT |
| S537A-RB1CC1-FLAG-R2 | GCGCGGATCCATGGAGCAGAAACTCATCTCTGAAGAGGATCTGATGAAGTTATATGTATTTCTGGTTA |
| S545A-RB1CC1-FLAG-F1 | GCGCGGTACCATGAAGTTATATGTATTTCTGGTTA |
| S545A-RB1CC1-FLAG-R1 | TCTTAAAAAAGCCTTCCTAA |
| S545A-RB1CC1-FLAG-F2 | TTAGGAAGGCTTTTTTAAGA |
| S545A-RB1CC1-FLAG-R2 | GCGCGGATCCATGGAGCAGAAACTCATCTCTGAAGAGGATCTGATGAAGTTATATGTATTTCTGGTTA |
| S537E-RB1CC1-FLAG-F1 | GCGCGGTACCATGAAGTTATATGTATTTCTGGTTA |
| S537E-RB1CC1-FLAG-R1 | AATTTCCCAAACTCTTCCCT |
| S537E-RB1CC1-FLAG-F2 | AGGGAAGAGTTTGGGAAATT |
| S537E-RB1CC1-FLAG-R2 | GCGCGGATCCATGGAGCAGAAACTCATCTCTGAAGAGGATCTGATGAAGTTATATGTATTTCTGGTTA |
| RB1CC1$^{\Delta C}$-FLAG-F | GCGCGGTACCATGAAGTTATATGTATTTCTGGTTA |
| RB1CC1$^{\Delta C}$-FLAG-R | GCGCGAATTCTCACTTATCGTCGTCATCCTTGTAATCAGATACTGAAGACATGCTCT |
| ELP3$^{WT}$-MYC-F | GCGCAAGCTTATGAGGCAGAAGCGGAAAGG |
| ELP3$^{WT}$-MYC-R | GCGCGAATTCCAGATCCTCTTCAGAGATGAGTTTCTGCTCTTATTTCAGCATCTTCACCA |

| | |
|---|---|
| ELP3$^{\Delta C}$-MYC-F | GCGCAAGCTTATGAGGCAGAAGCGGAAAGG |
| ELP3$^{\Delta C}$-MYC-R | GCGCGAATTCCAGATCCTCTTCAGAGATGAGTTTCTGCTCGGCTAGGATCCGAGCCACCA |

Primers for qPCR

| Name | 5'-3' |
|---|---|
| TPBG-qPCR-F | CTGAAGATGAGCGGCAGAACCGGAG |
| TPBG-qPCR-R | CAGTCGCAGACCCAGGGATTGTTGT |
| EPRS1-qPCR-F | TCATTGCCTGTCTCTGAGAACATAC |
| EPRS1-qPCR-R | GTGCCACTCGAGCTTTGGTTGTTGA |
| NAMPT -qPCR-F | AATCCAGGAAGCCAAAGATGTCTAC |
| NAMPT -qPCR-R | TGGCCACTGTGATTGGATACCAGGA |
| FOSL1-qPCR-F | TGGTGCCAAGCATCAACACCATGAG |
| FOSL1-qPCR-R | CCGGGCTGATCTGTTCACAAGGCCT |
| LZTFL1-qPCR-F | AGAGTTGGGCCTAAATGAGCA |
| LZTFL1-qPCR-R | CACAGCTTGTAATCCATTGAGGA |
| RB1CC1-qPCR-F | GAAAGAGCTTGCTCAGGGATT |
| RB1CC1-qPCR-R | TCATCAACTGATTTGCGTGACT |
| RB1-qPCR-F | CTCTCGTCAGGCTTGAGTTTG |
| RB1-qPCR-R | GACATCTCATCTAGGTCAACTGC |

| | |
|---|---|
| CHCHD3-qPCR-F | GAGGCGGACGAGAATGAGAAC |
| CHCHD3-qPCR-R | ACCAGAATACCGCTGAGACTTC |
| VRK3-qPCR-F | AAGTTCTCACTCAAACTGGATGC |
| VRK3-qPCR-R | TGGGGTCGAGTACAGCTTCTT |
| TPD52-qPCR-F | AGCATCTAGCAGAGATCAAGCG |
| TPD52-qPCR-R | AGCCAACAGACGAAAAAGCAG |
| SLC25A32-qPCR-F | TCCCCACACCGACAATATAAAGG |
| SLC25A32-qPCR-R | CCATGCGATGTTCCAAACAGC |
| MYC-qPCR-F | GTCAAGAGGCGAACACACAAC |
| MYC-qPCR-R | TTGGACGGACAGGATGTATGC |
| CYTH3-qPCR-F | GTGCTACGTGCTGTCATTCG |
| CYTH3-qPCR-R | TGGCGATGAACCGTTCTGC |
| MEPCE-qPCR-F | ACACCTGAGTATGATGTGGTGC |
| MEPCE-qPCR-R | GTAGATCCGGCGAAACATGC |
| CYREN-qPCR-F | AGGACTGTGTACTGCATGAATG |
| CYREN-qPCR-R | AACTTGTGTGAGGCGACACG |
| SEC13-qPCR-F | CCTGGCATCGTGCTCCTATG |
| SEC13-qPCR-R | GGATCAGGCCGTAGTCATGG |
| GAPDH-qPCR-F | GGAGCGAGATCCCTCCAAAAT |

| | |
|---|---|
| GAPDH-qPCR-R | GGCTGTTGTCATACTTCTCATGG |

Primers for ChIP-qPCR

| Name | 5'-3' |
|---|---|
| RBR-ChIP-F | CCGCCCAAGGAGGGAGAGTG |
| RBR-ChIP-R | CGGGCTGGGACGCTAAGTCA |
| NCR-ChIP-F | TCCTGCAAAGGACATGATCTCATTC |
| NCR -ChIP-R | GTAGGGATGGCAGGAAAGTGAAGGA |
| Chr3-p-ChIP-F | CAATCTCGCCAGCGTCTGAT |
| Chr3-p-ChIP-R | TTCCACAGTGGCTTCACTGA |
| Chr7-p1-ChIP-F | ACAGAGTGAGACTCCATCTC |
| Chr7-p1-ChIP-R | CTTCTTACTTGAGTTTCCTG |
| Chr7-p2-ChIP-F | ATCCATATGTACCAAATGAC |
| Chr7-p2-ChIP-R | TTCCTTAATGTGCATTTCTT |
| Chr8-p-ChIP-F | GTTTGGAAATAGTCTGGCAC |
| Chr8-p-ChIP-R | CTTTGGGACAATAGTTTGCA |
| Chr19-p-ChIP-F | GTAAGGCTACCAGGCACCAT |
| Chr19-p-ChIP-R | ATAGTTAACGGTAAGACTTA |

| | |
|---|---|
| ATP6V0A-ChIP-F | GTGGACTTTTCTCTGTGCAT |
| ATP6V0A-ChIP-R | CGTGGTGGTGCATGCCTGTA |

Primers for 3C assays

| Name | 5'-3' |
|---|---|
| Chr3/SEC13-3C-F | CTTCACAATCTCGCCAGCGTCTGAT |
| Chr3/SEC13-3C-R | CTTGGCCTTATCACCTAGAAAGTCA |
| Chr7p1/MEPCE-3C-F | TAAGGCAGGAGAATTGCTTGAATCT |
| Chr7p1/MEPCE-3C-R | CTGCCGCGCGATCCCACCCACCGTA |
| Chr7p1/CYREN-3C-F | TAAGGCAGGAGAATTGCTTGAATCT |
| Chr7p1/CYREN-3C-R | GGAGTCTGCAGGACCTGGCTGCGAA |
| Chr7p1/CHCHD3-3C-F | TAAGGCAGGAGAATTGCTTGAATCT |
| Chr7p1/CHCHD3-3C-R | CAGAATATACCCTTCACCTGGAATG |
| Chr7p2/CYTH3-3C-F | GTACTCAAGATTTTTAAGTGCCATA |
| Chr7p2/CYTH3-3C-R | AAGCATTTATAATTGATAGTATCTT |
| Chr8p/TPD52-3C-F | ACCAGTCATTGATGAATAGATGATG |
| Chr8p/TPD52-3C-R | GATGTTTATTTCTATACAATAGATCTC |
| Chr8p/SLC25A32-3C-F | CTGTTAAGACAGCACCTAGCACATA |
| Chr8p/SLC25A32-3C-R | TCACAAGACTCAATTATCTGTTTCA |

| | |
|---|---|
| Chr8p/MYC-3C-F | GTGCGTCTCCGAGATAGCAGGGGAC |
| Chr8p/MYC-3C-R | TCACAAGACTCAATTATCTGTTTCA |
| Chr19/VRK3-3C-F | CAGGTCCCCTTGGCACCAAGGCAAT |
| Chr19/VRK3-3C-R | GTATTGAGTACAGAGGTTTAGTATG |

Primers for luciferase reporter gene experiments

| Name | 5'-3' |
|---|---|
| WT-Chr3p-Luc-F | GCGCGGTACCCAATCTCGCCAGCGTCTGAT |
| WT-Chr3p-Luc-R | GCGCCTCGAGTTCCACAGTGGCTTCACTGA |
| WT-Chr7p1-Luc-F | GCGCGGTACCTGTAGTCCATGTACTCAAGA |
| WT-Chr7p1-Luc-R | GCGCCTCGAGAGCAAACAGCTGTTTGCTGC |
| WT-Chr7p2-Luc-F | GCGCGGTACCCTGGGTGACAGAGTGAGACT |
| WT-Chr7p2-Luc-R | GCGCCTCGAGCTTCTTACTTGAGTTTCCTG |
| WT-Chr8p-Luc-F | GCGCGGTACCCTTGCATCCAGGTAGGAGGT |
| WT-Chr8p-Luc-R | GCGCCTCGAGCATGCTCTCTAGGCAGGTAG |
| WT-Chr19p-Luc-F | GCGCGGTACCACAGTGAGTGATATGCAATG |
| WT-Chr19p-Luc-R | GCGCCTCGAGATCATGTATTGAGTACAGAG |
