# Supplementary material for: Tumour cells are sensitised to ferroptosis via RB1CC1‐mediated transcriptional reprogramming
Source: Clin Transl Med. 2022 Feb 27;12(2):e747. doi: 10.1002/ctm2.747 (PMC8882240; doi:10.1002/ctm2.747)
Supplement: Supplementary file 2 — Supplementary Table 2.pdf [file CTM2-12-e747-s001.pdf]

Supplementary Table 2. Basal information of patients in tissue microarray.

LUAD patient (N=194)		
Age		
	≥65	99
	<65	95
Gender		
	Male	100
	Female	94
Smoking		
	Yes	89
	No	105
Stage		
	I	65
	II	67
	III	62
Total		N=194

LUSC patient (N=154)		
Age		
	≥65	70
	<65	84
Gender		
	Male	86
	Female	68
Smoking		
	Yes	69
	No	85
Stage		
	I	45
	II	56
	III	53
Total		N=154
