# Supplementary material for: Tumour cells are sensitised to ferroptosis via RB1CC1‐mediated transcriptional reprogramming
Source: Clin Transl Med. 2022 Feb 27;12(2):e747. doi: 10.1002/ctm2.747 (PMC8882240; doi:10.1002/ctm2.747)
Supplement: Supplementary file 5 — Supplementary Table 5.0.docx [file CTM2-12-e747-s009.docx]

RB1CC1 phosphorylation mass spectrometry analysis results

| Group | Protein | Score | Amino acid | Peptide sequence | Position in the entire protein peptide sequence |
| --- | --- | --- | --- | --- | --- |
| DMSO | RB1CC1 | 129.76 | S | CLTRHSYRECLGRLDSLPEHEDSEKAEMKRS | S222 |
| DMSO | RB1CC1 | 89.663 | S | SLPEHEDSEKAEMKRSTELVLSPDMPRTTNE | S237; T238 |
| DMSO | RB1CC1 | 89.642 | T | SASSPRMESTAGITTTTSPRTPPPLTVQDPL | T663; T664; T665; T666; S667 |
| erastin | RB1CC1 | 120.6 | S | CLTRHSYRECLGRLDSLPEHEDSEKAEMKRS | S222 |
| erastin | RB1CC1 | 109.16 | S | SLPEHEDSEKAEMKRSTELVLSPDMPRTTNE | S237; T238 |
| RSL3 | RB1CC1 | 195.6 | S | SQTITDLLSEQKASVSQTSPQSASSPRMEST | S644 |
| RSL3 | RB1CC1 | 161.82 | S | ITDLLSEQKASVSQTSPQSASSPRMESTAGI | T646; S647; S650 |
| RSL3 | RB1CC1 | 94.007 | S | SSPRMESTAGITTTTSPRTPPPLTVQDPLCP | T663; T664; T665; T666; S667 |
| RSL3 | RB1CC1 | 94.747 | S | SLPEHEDSEKAEMKRSTELVLSPDMPRTTNE | S237; T238 |
