# Supplementary material for: Tumour cells are sensitised to ferroptosis via RB1CC1‐mediated transcriptional reprogramming
Source: Clin Transl Med. 2022 Feb 27;12(2):e747. doi: 10.1002/ctm2.747 (PMC8882240; doi:10.1002/ctm2.747)
Supplement: Supplementary file 10 — 2022‐0204Revision supplementary figures legends.docx [file CTM2-12-e747-s004.docx]

**Supplementary Figures 1-7**

**Supplementary Figure. 1
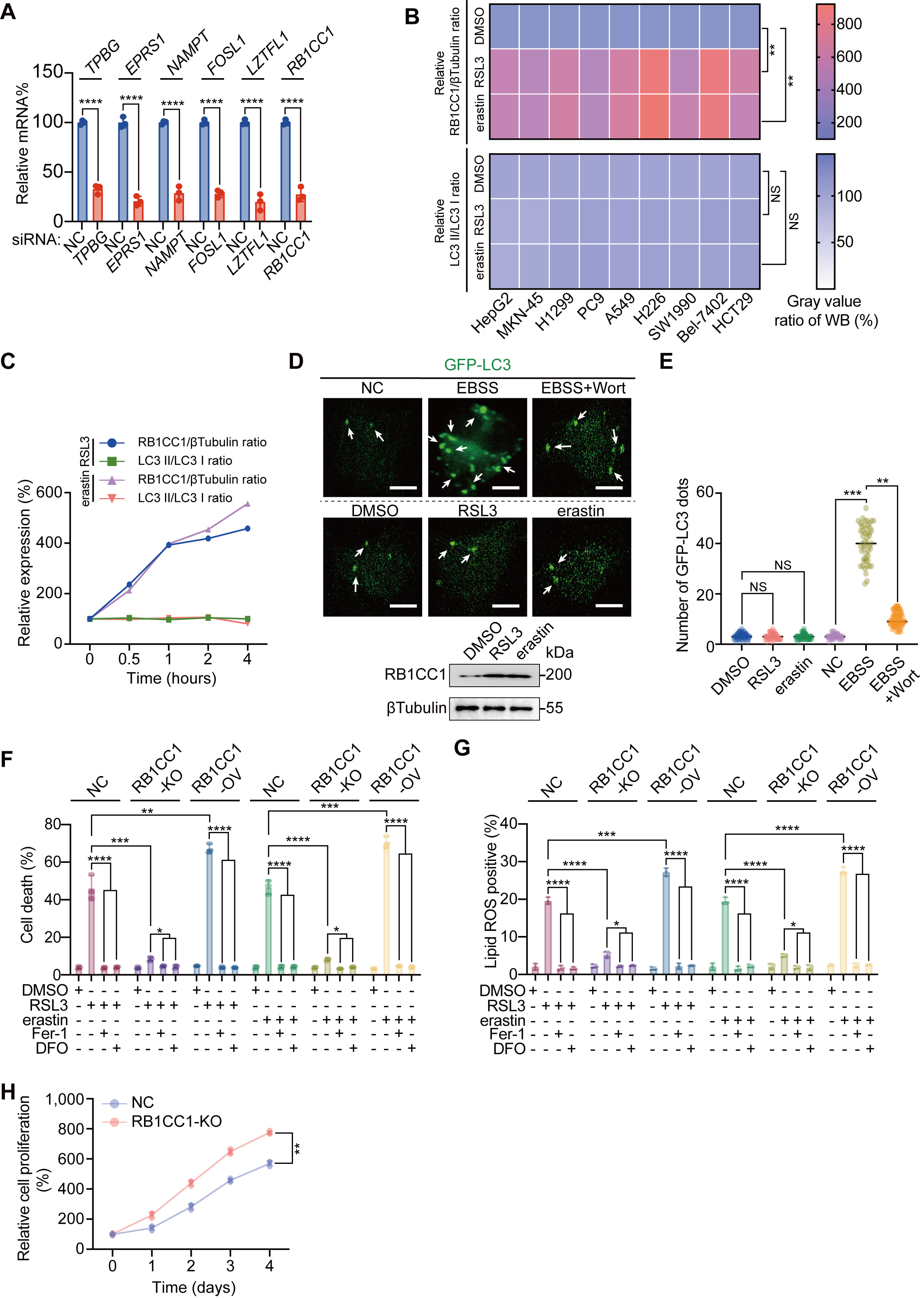
**

**Supplementary Figure. 1. Supplementary to Figure. 1.**

(A) siRNA efficiency of genes, as indicated, in HepG2 cells, as measured by qPCR.

(B) Relative RB1CC1/βTubulin and LC3 II/LC3 I ratios in different cell lines, as indicated, treated with RSL3 (1 µM) or erastin (10 µM) for 4h. The representative IB images are shown in Fig. 1C.

(C) Relative RB1CC1/βTubulin and LC3 II/LC3 I ratios in HepG2 cells treated with RSL3 (1 µM) or erastin (10 µM) for indicated hours. The representative IB images are shown in Fig. 1D.

(D) Autophagosome formation was monitored by fluorescence microscopy in HepG2 cells transiently transfected with GFP-LC3 expression plasmid following treating with RSL3 (1 µM) or erastin (10 µM) for 2h. Treating EBSS was regarded as a positive control. Wortmannin was treated at a final concentration of 50nM. Scale bar, 10 µm. RB1CC1 was examined by IB at the same time.

(E) The autophagosome was counted from 50 cells for each group in Supplementary Fig. 1D.

(F-G) Cell death (F) and lipid ROS generation (G) in control and HepG2 cells with RB1CC1 KO or OV, treating with RSL3 (1 µM) or erastin (10 µM), in the presence or absence of Fer-1 (2 µM) or DFO (100 µM) for 12h.

(H) Cell proliferation of control cells and RB1CC1-KO cells.

Statistical analysis was performed by Student’s t-test (A, B, E, F, G) or one-way ANOVA (F, G) or Two-way ANOVA (H). Data are presented as means ± SD from indicated samples. ****p < 0.0001, ***p < 0.001, **p < 0.01, *p<0.05, indicates statistical significance and N.S. indicates non-significance.

**Supplementary Figure. 2**

**
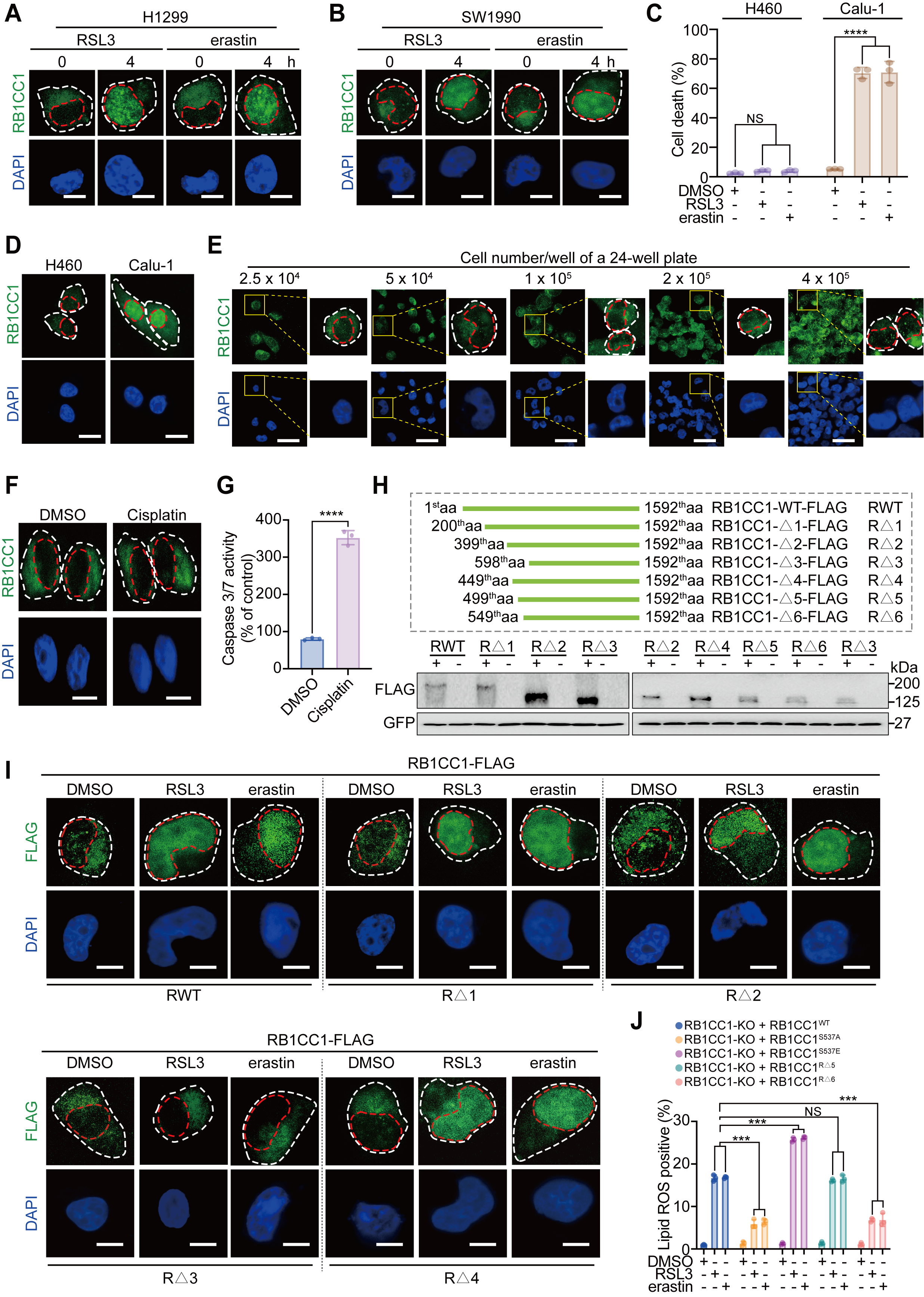
**

**Supplementary Figure. 2. Supplementary to Figure. 2.**

(A-B) Subcellular localization of RB1CC1 in H1299 (A) and SW1990 (B) cells treating with RSL3 (1 µM) or erastin (10 µM) for indicated time. Scale bar, 10 µm.

(C) Cell death in H460 and Calu-1 cells following treating with DMSO, RSL3 (1 µM) or erastin (10 µM) for 12h.

(D) Subcellular localization of RB1CC1 in H460 and Calu-1 cells. Scale bar, 10 µm.

(E) Subcellular localization of RB1CC1 in HepG2 cells under different cell density. Scale bar, 50 µm.

(F-G) Subcellular localization of RB1CC1 and caspase 3/7 activity in HepG2 cells following treating with DMSO, cisplatin (5 µM) for 12h.

(H) Schematic presentation of truncated version of RB1CC1-FLAG. The expression of RB1CC1 was also verified by IB.

(I) The subcellular localization of indicated truncated version of exogenous RB1CC1 in HepG2 cells treated with DMSO, RSL3 (1 µM) or erastin (10 µM) for 4h. Scale bar, 10 µm.

(J) Lipid ROS generation was measured in RB1CC1-KO HepG2 cells reconstituted with WT-, S537A-, S537E-, RΔ5-, RΔ6-RB1CC1 constructs following treating with DMSO, RSL3 (1 µM) or erastin (10 µM) for 12h.

Statistical analysis was performed by one-way ANOVA (C, G, J). Data are presented as means ± SD from indicated samples. ****p < 0.0001, ***p < 0.001, indicates statistical significance and N.S. indicates non-significance.

**Supplementary Figure. 3**


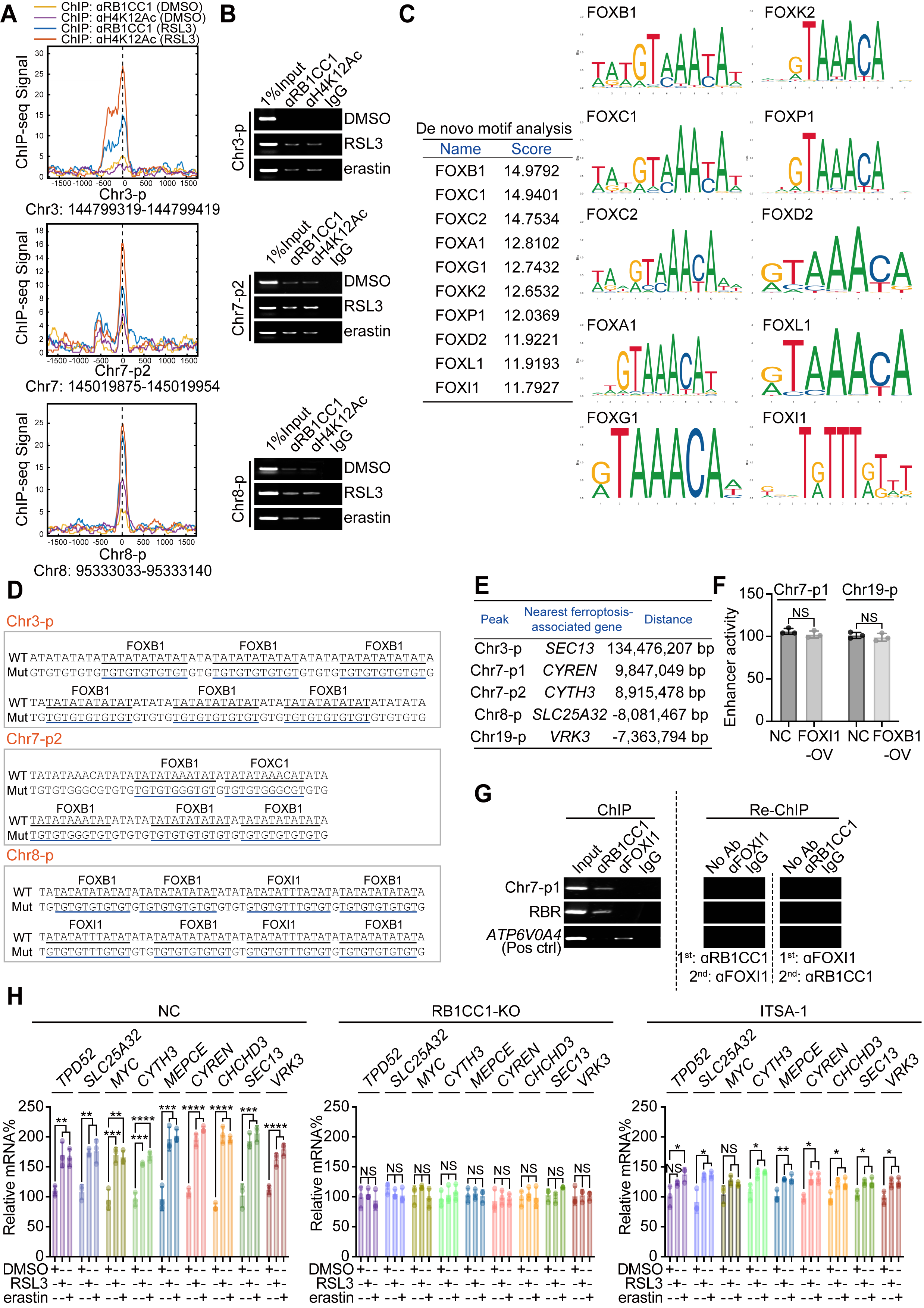


**Supplementary Figure. 3. Supplementary to Figure. 3.**

(A) Presentation of ChIP-seq peaks for Chr3-p, Chr7-p2 and Chr8-p in HepG2 cells treated with DMSO or RSL3 (1 µM) for 4h.

(B) Verification of RB1CC1 binding and H4K12Ac modification within the Chr3-p, Chr7-p2 and Chr8-p peaks in HepG2 cells treated with DMSO, RSL3 (1 µM) or erastin (10 µM) for 4h.

(C) De novo motif analysis showing the existence of FOX family member motifs within ChIP-seq peaks that overlapped by RB1CC1 and H4K12Ac.

(D) Sequences of Chr3-p, Chr7-p2 and Chr8-p with WT or mutant FOX motif.

(E) The ferroptosis-associated genes nearest the ChIP-seq peaks and the distances between them.

(F) Enhancer activities of Chr7-p1 and Chr19-p in HepG2 cells with or without FOXI1 or FOXB1 overexpression.

(G) ChIP and Re-ChIP showing the binding status of RB1CC1 and FOXI1 at the Chr7-p1 and *RB1* promoter. The *ATP6V0A4* locus was parallel examined as the positive control for FOXI binding.

(H) The expression of ferroptosis-associated genes, as measured by qPCR in control HepG2 cells, RB1CC1-KO HepG2 cells and HepG2 cells pretreated with ITSA-1 (50 µM, 2h), in the presence or absence of DMSO, RSL3 (1 µM) or erastin (10 µM) for 4h.

Statistical analysis was performed using Student’s t-test (F) or one-way ANOVA (H). Data are presented as means ± SD from indicated samples. ****p < 0.0001, ***p < 0.001, **p < 0.01, *p<0.05, indicates statistical significance and N.S. indicates non-significance.

**Supplementary Figure. 4**


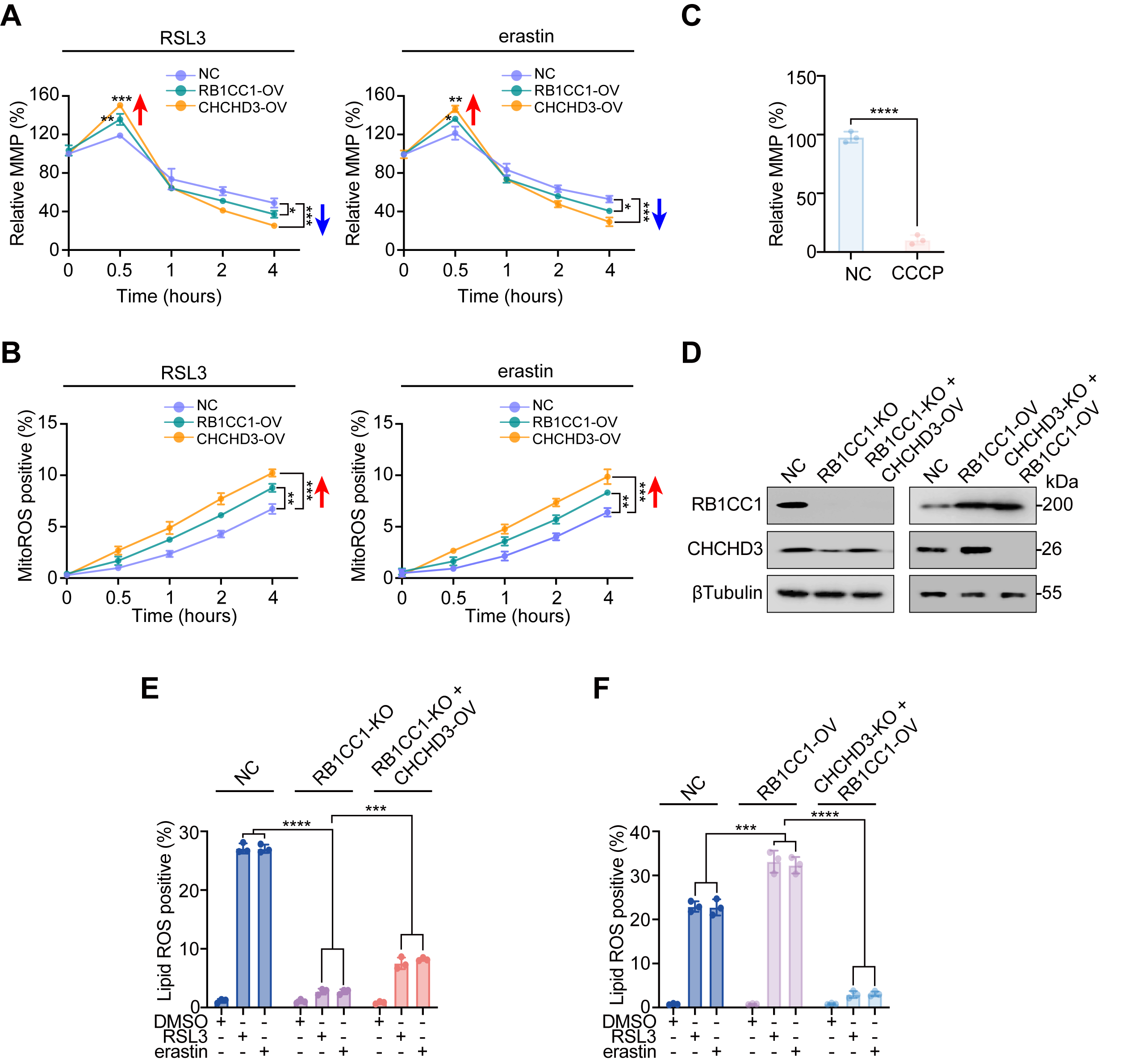


**Supplementary Figure. 4. Supplementary to Figure. 4.**

(A) Dynamic MMP alteration in control, and HepG2 cells with RB1CC1 or CHCHD3 OV, under the treatment of RSL3 (1 µM) or erastin (10 µM) for indicated time.

(B) Dynamic mitoROS alteration in control, and HepG2 cells with RB1CC1 or CHCHD3 OV, under the treatment of RSL3 (1 µM) or erastin (10 µM) for indicated time.

(C) MMP before and after treating with CCCP (10 µM, 1h) in HepG2 cells.

(D) RB1CC1 and CHCHD3 in HepG2 cells under indicated treatment, as measured by IB.

(E) Lipid ROS generation in control and HepG2 cells with RB1CC1 KO, with or without CHCHD3 OV, under the treatment of DMSO, RSL3 (1 µM) or erastin (10 µM) for 12h.

(F) The effects of RB1CC1 OV on lipid ROS generation, as measured in HepG2 cells with or without CHCHD3 KO, in the presence or absence of RB1CC1 OV, under the treatment of DMSO, RSL3 (1 µM) or erastin (10 µM) for 12h.

Statistical analysis was performed using Two-way ANOVA (A, B) or Student’s t-test (C) or one-way ANOVA (E, F). Data are presented as means ± SD from indicated samples. ****p < 0.0001, ***p < 0.001, **p < 0.01, *p < 0.05, indicates statistical significanc.

**Supplementary Figure. 5**


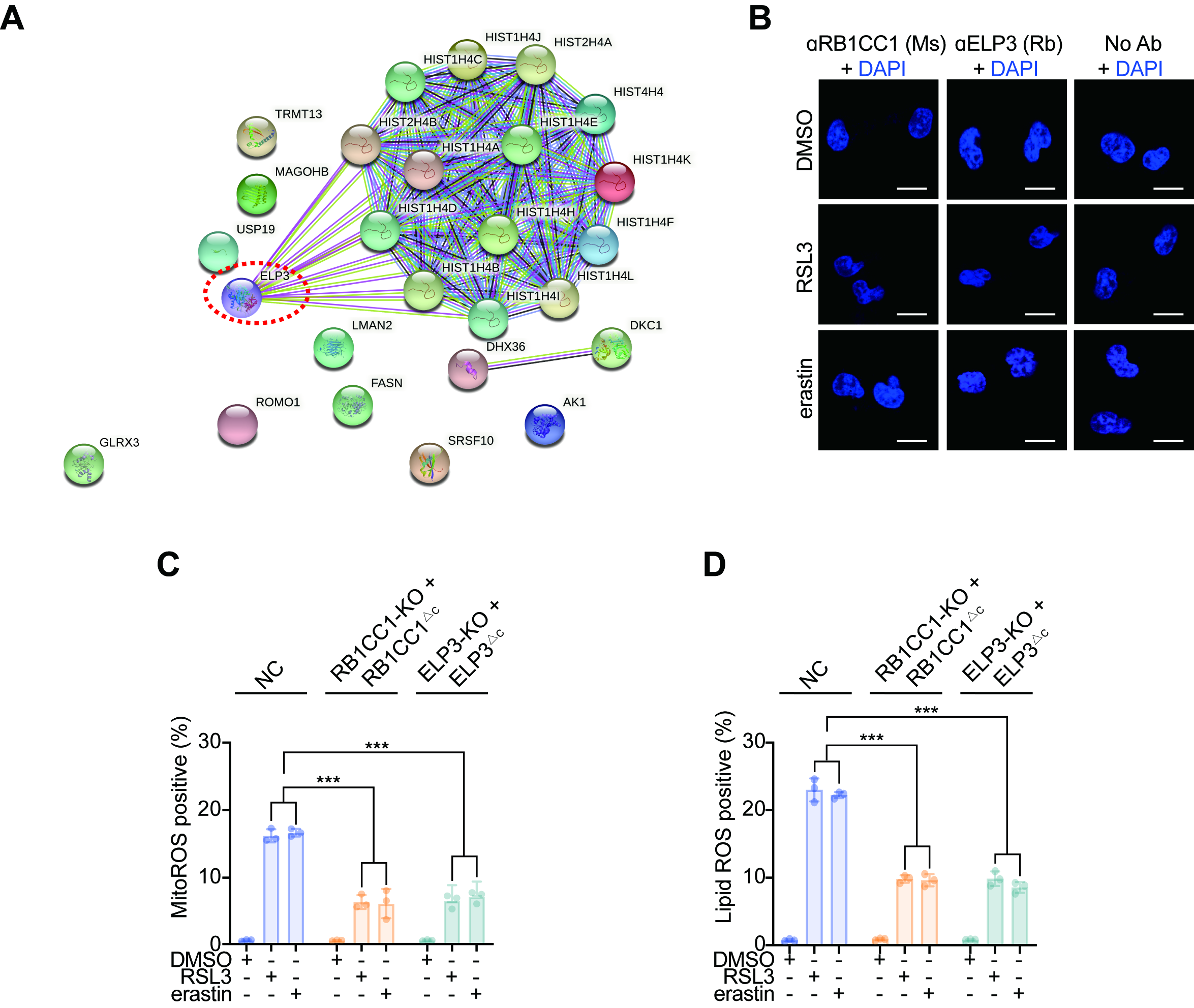


**Supplementary Figure. 5. Supplementary to Figure. 5.**

(A) STRING demonstrated protein-protein interactions between H4 and ferroptosis-associated proteins, as predicted in Fig. 5A.

(B) PLA results in HepG2 cells when only anti-RB1CC1 and anti-ELP3 antibodies were used. Scale bar, 10 µm.

(C-D) MitoROS (C) and lipid ROS generation (D) in control cells, RB1CC1-KO and ELP3-KO HepG2 cells reconstituted with RB1CC1^△C^-FLAG and ELP3^△C^-Myc, under the treatment of DMSO, RSL3 (1 µM) or erastin (10 µM) for 12h.

Statistical analysis was performed using one-way ANOVA (C, D). Data are presented as means ± SD from indicated samples. ***p < 0.001, indicates statistical significance.

**Supplementary Figure. 6**


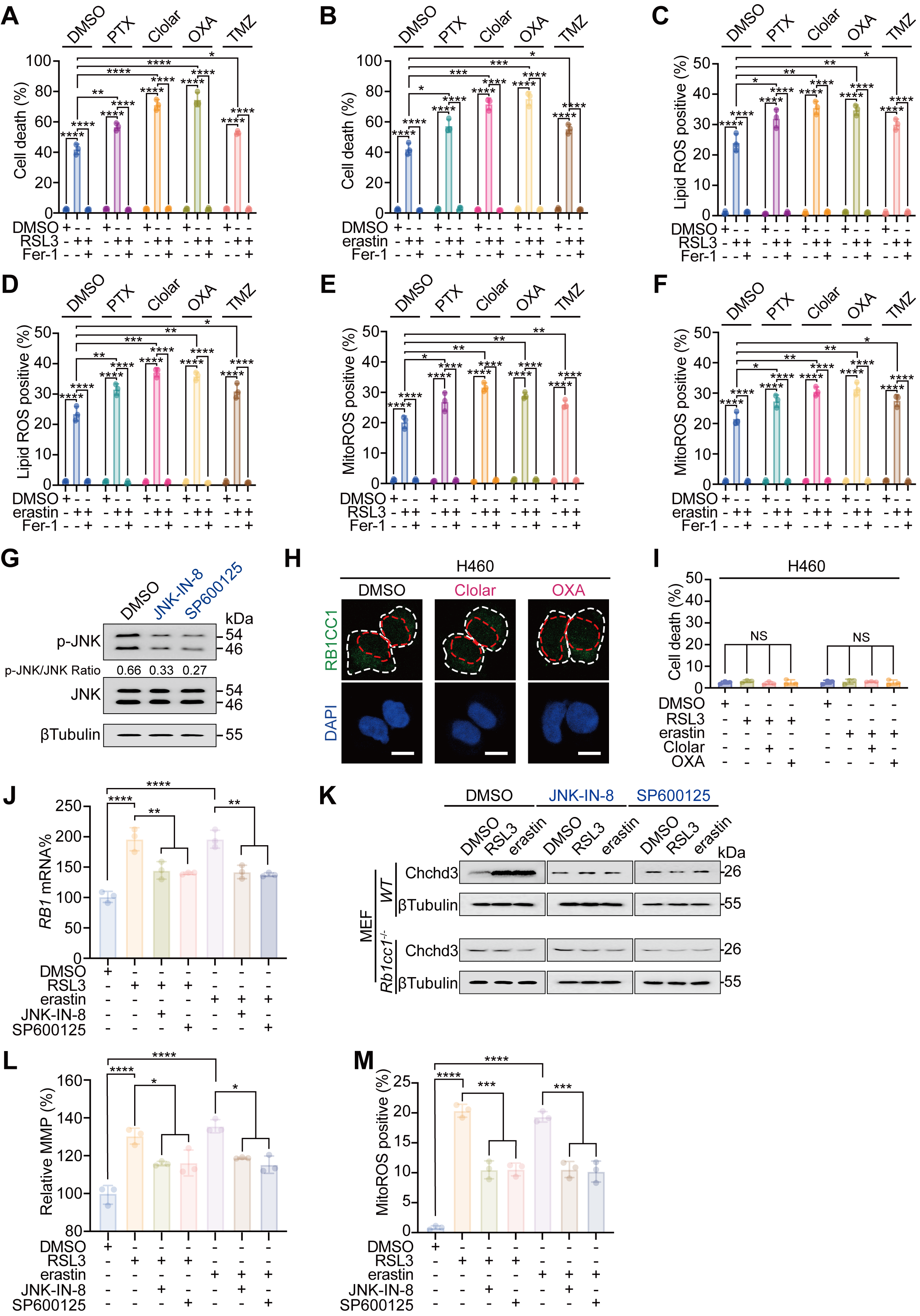


**Supplementary Figure. 6. Supplementary to Figure. 6.**

(A-F) Cell death (A-B), lipid ROS (C-D) and mitoROS (E-F) generation were measured in A549 cells treated with DMSO, RSL3 (1 µM), erastin (10 µM), Fer-1 (2 µM), PTX (50 pM), Clolar (10 µM), OXA (50 µM) or TMZ (50 µM), as indicated for 12h.

(G) Phosphorylation of JNK in A549 cells treated with DMSO, JNK-IN-8 (1 µM) or SP600125 (10 µM) for 1h.

(H) Subcellular localization of RB1CC1 in H460 cells treating with DMSO, Clolar (10 µM), OXA (50 µM) for 12h. Scale bar, 10 µm.

(I) Cell death were measured in H460 cells treated with DMSO, RSL3 (1 µM) or erastin (10 µM) for 12h, in the presence or absence of Clolar (10 µM) or OXA (50 µM).

(J) *RB1* mRNA in A549 cells treated with DMSO, RSL3 (1 µM) or erastin (10 µM) for 4h, in the presence or absence of JNK-IN-8 (1 µM) or SP600125 (10 µM).

(K) Chchd3 in *WT* or *Rb1cc1*^-/-^ MEF cells treated with DMSO, RSL3 (1 µM) or erastin (10 µM), in the presence or absence of JNK-IN-8 (1 µM) or SP600125 (10 µM) for 1h.

(L-M) MMP (J) and mitoROS (K) in A549 cells treated with DMSO, RSL3 (1 µM) or erastin (10 µM) for 0.5h (J) or 12h (K), in the presence or absence of JNK-IN-8 (1 µM) or SP600125 (10 µM).

Statistical analysis was performed using Student’s t-test (A, B, C, D, E, F, I, J, L, M). Data are presented as means ± SD from indicated samples. ****p < 0.0001, ***p < 0.001, **p < 0.01, *p<0.05, indicates statistical significance and N.S. indicates non-significance.

**Supplementary Figure. 7**


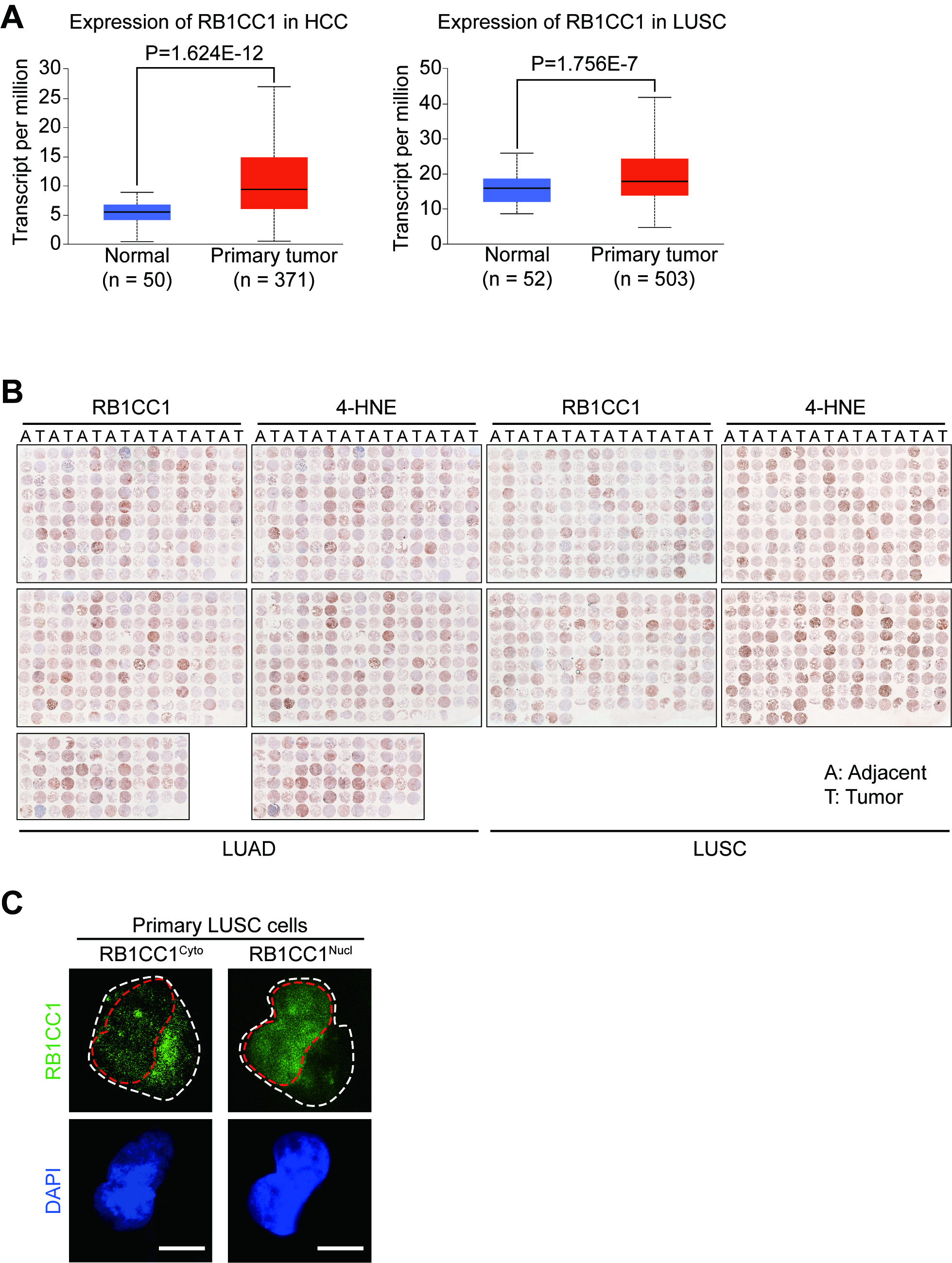


**Supplementary Figure. 7. Supplementary to Figure. 7.**

(A) The expression of RB1CC1 in normal, HCC and LUSC tissues, as analyzed using data from UALCAN.

(B) TMA images of RB1CC1 and 4-HNE in LUAD and LUSC.

(C) Subcellular localization of RB1CC1 in primary LUSC cells. Scale bar, 10 µm.
